# Addressing cultural, racial and ethnic discrepancies in guideline discordant gestational weight gain: a systematic review and meta-analysis

**DOI:** 10.7717/peerj.5407

**Published:** 2018-08-27

**Authors:** Kathryn M. Denize, Nina Acharya, Stephanie A. Prince, Danilo Fernandes da Silva, Alysha L.J. Harvey, Zachary M. Ferraro, Kristi B. Adamo

**Affiliations:** 1 School of Human Kinetics, Faculty of Health Science, University of Ottawa, Ottawa, Canada; 2 Division of Cardiac Prevention and Rehabilitation, University of Ottawa Heart Institute, Ottawa, Canada; 3 Department of Physical Education, State University of Midwest/Parana (UNICENTRO), Guarapuava, Paraná, Brazil; 4 Faculty of Medicine, University of Ottawa, Ottawa, Canada

**Keywords:** Culture, Ethnicity, Gestational weight gain, Institute of medicine, Meta-analysis, Pregnancy, Race, Systematic review

## Abstract

**Objective:**

To systematically review the literature and describe the discrepancies in achieving the 2009 Institute of Medicine (IOM) gestational weight gain (GWG) guidelines across cultures.

**Methods:**

Ten databases were searched from inception to April 2018. Observational cohort studies were included that examined adult women; reported on a measure of culture; compared cultural groups, and reported on GWG. Articles were**** broken down into papers that used the current 2009 IOM GWG guidelines and those that used others. A meta-analysis was conducted for studies using the 2009 guidelines examining the prevalence of discordant GWG across cultural groups.

**Results:**

The review included 86 studies. Overall, 69% of women experienced discordant GWG irrespective of culture. White women experienced excessive GWG most often, and significantly more than Asian and Hispanic women; Black women had a higher prevalence of excessive GWG than Hispanic and Asian women; however, this difference was not significant.

**Conclusions:**

The majority of women experience excessive GWG, with White women experiencing this most often. Culturally diverse GWG guidelines are needed to individualize antenatal care and promote optimal maternal-fetal health outcomes across cultural groups.

## Introduction

Pregnancy is a critical period as a mother’s health can be a strong indicator of her child’s health (Adamo, Ferraro & Brett, 2012; Prather, Spitznagle & Hunt, 2012; Rauh et al., 2014). Gestational weight gain (GWG) has repeatedly shown to be a robust predictor of adverse health outcomes; including the perpetuation of the intergenerational cycle of obesity (i.e., fetal overgrowth, high post-partum weight retention, subsequent obesity in mother and infant) (Ferraro et al., 2012; Egan et al., 2014; Gaudet et al., 2014; Diesel et al., 2015; Van Rossem et al., 2015; Baugh et al., 2016). Additional adverse health risks associated with excessive GWG include a greater risk of hypertension (Egan et al., 2014; Baugh et al., 2016; Chasan-Taber et al., 2016) in mothers and higher blood pressure in children (Gaillard et al., 2015). In contrast, inadequate GWG has been linked to premature birth and small-for-gestational-age (SGA) infants (Mumbare et al., 2012; Baugh et al., 2016).

In attempts to minimize risk to mom and baby, and achieve the best health outcomes, the Institute of Medicine’s (IOM) 2009 guidelines classifies GWG adequacy by pre-pregnancy BMI (Rasmussen & Yaktine, 2009). Despite the availability of these guidelines, only 30–40% of women are reported to gain within the recommended range; with most exceeding the guidelines (McDonald et al., 2011). Predictors of GWG include: pre-pregnancy weight; (Masho, Bishop & Munn, 2013; Rosal et al., 2016) socioeconomic status (SES); maternal health behaviours; (Ota et al., 2011; Heery et al., 2015) maternal age and parity (Vahratian, 2009). To date, the associations reported between race/ethnicity and discordant GWG have been diverse,(Shieh & Wu, 2014; Liu et al., 2014) likely a result of the variability in the definition of race/ethnicity and the social contexts in which they are examined. The revised 2009 IOM guidelines identified culture as a determinant of GWG (Rasmussen & Yaktine, 2009) but lacked systematic review evidence. Culture largely evades definition, with little consensus among experts (Dykstra, 2009). Therefore, the use of race/ethnicity alone is not adequate to characterize the multitude of factors that comprise a culture’s impact on pregnancy. This systematic review, therefore, attempts to encompass the various aspects of culture including race, ethnicity, language, nationality, and acculturation. Race and ethnicity are historically salient contributors to culture,(Xifra & McKie, 2011); however, due to increasing globalization and migration, other factors such as language, nationality, and acculturation (adoption of values and customs of other groups due to immigration) must be considered when identifying and characterizing different cultures (Fuligni et al., 2008).

Although a previous review by Headen et al. (2012) examined the associations between racial/ethnic identities and GWG, it was narrative in nature and limited in its inclusion criteria. The Headen review focused on White, Black and/or Hispanic women within the United States (US), compared GWG to the 1990 IOM guidelines, and excluded pregnancies complicated by adverse maternal-fetal health outcomes. The conclusion of their review identified that greater research surrounding the social context of race and GWG was needed (Headen et al., 2012). Therefore, the purpose of the present systematic review was to examine the discrepancies in achieving the updated 2009 IOM guidelines across cultures (more broadly represented by race, nationality, ethnicity, language and immigration status).

## Methods

### Sources

This systematic review was prospectively registered in the PROSPERO database (#CRD42015023399) and the protocol published elsewhere (Manyanga et al., 2015). This systematic review was conducted in accordance with the Preferred Reporting Items for Systematic Reviews and Meta-Analysis (PRISMA) guidelines (Moher et al., 2010). Search strategies were developed with two Health Science Librarians and searches were performed by KD and NA. Ten bibliographic databases were searched including: Ovid MEDLINE; EMBASE; Clinicaltrials.gov; Cochrane Central Register of Controlled Trials; CINAHL; PsycINFO; Sociological Abstracts; Literature Latino-Americana e do Caribe em Ciencias da Saude (LILACS), IBECS; and, Cuba Medicina (CUMED). The search strategy used for Ovid MEDLINE is presented in Table S1 and was modified according to the indexing parameters for each database. The Ovid interface was used to search MEDLINE, EMBASE, Cochrane Central Register of Controlled Trials, and PsycINFO. CINAHL was searched using EBSCOhost, Sociological Abstracts using Proquest, and the Virtual Health Library Regional Portal was used to search LILACS, IBECS and CUMED. Canadian Agency for Drugs and Technologies in Health (CADTH’s) Grey matters and citations of relevant systematic reviews and trials were also hand searched. The search was initially run first from database inception up to July 7, 2015; it was then updated to include articles published until April 2018, inclusively. The original search yielded 3,628 articles and the update added 1,058 articles to the initial screening.

### Study selection

#### Population

Studies with the majority (≥80%) of participants being adult (≥18 years of age) pregnant women were included.

#### Exposure

Culture was the exposure; which was broadly defined by ethnicity/nationality/race/language/immigration status. Studies were included if they reported at least one of these determinants.

#### Comparators

Studies were included if they compared at least two different cultural groups. When a study looked at the outcome in only one cultural population, it was excluded.

#### Outcomes

The primary outcome was inadequate or excessive GWG (hereafter referred to as discordant GWG), as defined by the IOM. Studies that used the 1990 guidelines were included but analyzed separately from studies that compared GWG using the updated 2009 guidelines. Secondary outcomes include maternal-fetal health outcomes such as large-for-gestational-age (LGA), macrosomia, gestational diabetes mellitus (GDM), and all pregnancy-induced hypertension disorders.

#### Study design

This review systematically identified prospective and retrospective observational and cohort studies. The language of publication was not an exclusion factor, and relevant translation was procured as necessary (NA and DFdS).

Several updates were made to the published protocol. Randomized controlled trials (RCTs) were included in the original inclusion criteria; however, the study design was unable to appropriately address the research question and was excluded hereafter. While the GRADE approach to quality assessment (Balshem et al., 2011) was described in the initial protocol, it was not used due to the ineligibility of RCTs. Retrospective studies were not part of the original inclusion criteria but were included to appropriately answer our research question and to yield a greater sample size. While “culture” and “ancestry” were included in the original search criteria, these terms were difficult to quantify. If ancestry was reported, it was generally classified under ethnicity. Instead, factors such as race, ethnicity, nationality, and language were used to distinguish different cultures in the selected articles. Language was added as a potential measure of culture as it can be reflective of acculturation. This association has been recently shown by Boone et al. who found a correlation between time spent in the US and mastery of the English language (Boone et al., 2007).

The results of the search were imported into Covidence (Cochrane, Melbourne, Australia), and duplicates were removed before initial screening. Two independent reviewers (original search: TM and DfdS, update: NA and KD) screened the titles and abstracts of the search results and marked each as ‘include’, ‘exclude’ or ‘unsure’ based on the eligibility criteria. The full texts of the studies classified as ‘unsure’ or ‘include’ were then reviewed by the same two reviewers based on each of the eligibility criteria. Conflicts were resolved through consensus and discussion with a third reviewer (ZMF).

Standardized data collection forms were created and tested on a sample of studies. After changes were made, data extraction was carried out for each article by two independent reviewers. Reviewers were not blinded to authors or study titles. Conflicts were resolved as described above. Separate extraction sheets were used for studies that applied the 2009 IOM guidelines (Rasmussen & Yaktine, 2009) and those using ‘other’ guidelines. Extracted data included: country of study; country income level; region; time of data collection; funding source; inclusion criteria and exclusion criteria; study design; follow-up length; number of study centers; study setting; primary outcome(s); and, direction of association between culture and discordant GWG. Studies that used 2009 IOM guidelines were reviewed quantitatively and had the following additional information extracted for each cultural group defined: number of participants; mean/median age; socioeconomic covariates (highest level of education, mean household income); number of smokers; pre-pregnancy weight/body mass index (BMI) classification; cultural variables (e.g., ethnicity, race, nationality, language, immigration status); and total GWG and classification as ‘inadequate’, ‘adequate’, and ‘excessive’ as per the IOM guidelines. Perinatal and neonatal outcomes such as GDM, LGA, SGA, hypertension, macrosomia, and mode of delivery were also captured in the extraction.

When five or more studies were available to describe the rates of GWG under a specific racial/ethnic group, a meta-analysis was conducted. Four racial/ethnic groups were identified as having a sufficient number of studies: ‘White’; ‘Black’; ‘Hispanic’; and, ‘Asian’. White, Caucasian and non-Hispanic White women; and Black, African American, and non-Hispanic Black women were grouped into ‘White’ and ‘Black’, respectively. Analyses were conducted by comparing these four racial/ethnic groups.

Meta-analyses were completed to compare the proportion (and 95% confidence intervals [CIs]) of women in each study who experienced excessive or inadequate GWG within a racial/ethnic group. A random-effects meta-analysis was conducted to provide an overall measure of effect (proportion with excessive/inadequate GWG) and 95% CIs for each population group (i.e., White, Black, Hispanic, Asian). Cochrane’s Q statistic and the *I*^2^ statistic were used to assess heterogeneity between studies. Heterogeneity was classified as low (25%), moderate (50%), or high (75%) (Higgins et al., 2003). Forest plots were created using an Excel template (Neyeloff, Fuchs & Moreira, 2012), and publication bias was assessed using Egger’s tests with Meta-Essentials software (Suurmond, Van Rhee & Hak, 2017). Statistical significance was set at *p* < 0.05. Studies that characterized culture by language, nationality, or acculturation were included in a narrative analysis. A subgroup analysis was performed to identify sources of heterogeneity when *I*^2^ >50%; various *a priori* methodological (quality; low vs. high bias, number of participants, and study design; retrospective vs. prospective) and clinical (population; healthy vs. women with pregnancy-related complications, region; the US vs. other, and center; single vs. multi) variables were investigated. The review differed from the published protocol as other *a priori* subgroups analyses were unfeasible due to inadequate reporting or lack of studies. A large portion of studies utilized ’other’ or no guidelines; this data was synthesized and analyzed separately.

Data were also extracted related to *a priori* secondary outcomes including weight loss, GDM, gestational hypertension, pre-eclampsia, mode of delivery, length of stay in hospital, LGA, SGA, shoulder dystocia, and prematurity. When two or more studies compared the same cultural group for a given variable, they were analyzed and reported.

A modified Cochrane Risk of Bias Tool was used to assess the level of bias in each study included in the quantitative analysis. The Tool was modified for use in the evaluation of bias in prospective and retrospective study designs (Poitras et al., 2016). Given the large number of retrospective cohort studies identified, this tool was most suited to appropriately determine the risk of bias in the selected studies. Each study was classified as having high, low or unclear risk of the following biases: selection bias (how participants are selected to be in the study); performance bias (flawed measurement of exposure); detection bias (flawed measurement of outcome); reporting bias (selective outcome reporting); attrition bias (incomplete follow-up; high loss to follow-up); and, other bias (other factors: control for confounding variables, sample represents population).

## Results

**Figure 1:**
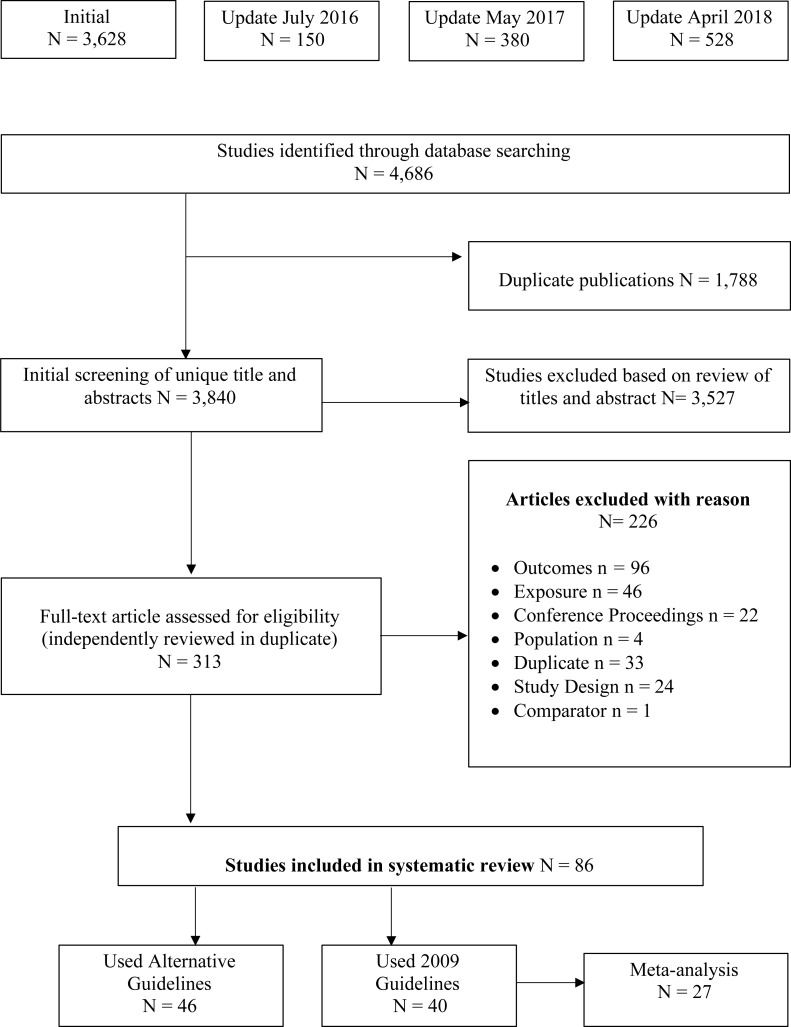
PRIMSA flow diagram of study selection process. An overview of the study selection process, including the original search and three subsequent updated searches. Reasons for article exclusion are provided.

An outline of the study identification, inclusion and exclusion process is outlined in Fig. 1. In total, 4,686 titles and abstracts were screened. Of these, 313 articles met the criteria for full-text screening. Overall, 86 (81 unique samples) papers were identified as meeting the inclusion criteria and were included in the review.

**Table 1:** Study characteristics of papers using the 2009 IOM guidelines listed in alphabetical order, by author.

**Author, year**	**Country of study**	**N**_**analyzed**_	**Population description**	**Maternal age, years (mean ± SD or range [%])**	**Racial/ethnic groups**
Badreldin, Grobman & Pool (2018)	US	29,380	General population	Weight loss: 28.6 ± 1.48	NHW, Black, Hispanic, Asian
				Inadequate GWG: 31.0 ± 5.9	
				Adequate GWG: 31.9 ± 5.1	
				Excessive GWG: 31.4 ± 5.4	
Berggren, Stuebe & Boggess (2015)	US	466	Women who were diagnosed with GDM	Inadequate GWG: 32.0 ± 5.3	Black, White, Hispanic
				Adequate GWG: 31.3 ± 5.5	
				Excessive GWG: 30.5 ± 6.0	
Bodnar et al. (2001)	US	23,362	General population	<20: 8%	NHW, NHB
				≥20: 92%	
Bogaerts et al. (2012)	Belgium	54,022	General population	<20: 2%	Belgian, Dutch, Turkish, Moroccan
				20–29: 52%	
				>30: 46%	
Bowers et al. (2013)	US	105,985	General Population	LGA: 28.65 (5.8)	NHW, NHB, Hispanic, Asian
				Macrosomia (>4,000 g): 28.5 ± 5.7	
				Macrosomia (>4,500 g): 29.4 ± 20.0	
				Normal weight: 27.2 ± 5.8	
Cavicchia et al. (2014)	US	132,574	General population	<20: 15%	NHW, NHB, Hispanic
				20–34: 75%	
				>35: 10%	
Chaffee et al. (2015)	US	4,780	General population	23.8 ± 5.5	Non-Hispanic Non-Black, NHB, Hispanic Non-Black
Chang et al. (2017)	US	1,034	High risk population	20.6 ± 3.0	Black, White, Hispanic
Chasan-Taber et al. (2016)	US	1,359	General population	<19: 31.4%	Born in Puerto Rico/Dominican Republic, Born in the US and low and high acculturation
				19–23: 39.2%	
				24–29: 17.5%	
				>30: 11.8%	
Cheng et al. (2015)	US	114,632	General population	Majority <35	NHW, Asian
Chihara et al. (2014)	US	19,130	General population	<20: 14%	White, Asian, Hawaiian, Pacific Islander
				20–29: 62%	
				>30: 24%	
Cohen et al. (2016)	US	6,344	General population	25.4 ± 5.1	White/Other, Black/African American, Hispanic/Latina
Cox Bauer et al. (2016)	US	10,734	Women who are obese (BMI ≥ 30 kg/m^2^)	28.9 ± 7.93	NHW, NH Other, Hispanic/Latina
Deputy et al. (2015)	US	44,421	General population	NR	White, Black, Hispanic, Asian, Native American, Alaskan Native, Hawaiian, Other
Fontaine et al. (2012)	US	2,760	General population	28.1 ± 5.3	Black, White
Rothberg et al. (2011)	US	427	General population	Underweight: 20.5 ± 2.4	Black, Hispanic, Caucasian, Other
				Normal weight: 20.6 ± 2.8	
				Overweight: 21.0 ± 2.6	
				Obese: 21.1 ± 2.5	
Haile et al. (2017)	US	2,053	General population	18–24: 19.3%	NHW, NHB, Hispanic
				25–34: 64.2%	
				>35: 16.5%	
Harris et al. (2015)	US	856	General Population	<20: 14%	NHW, NHB, Other
				20–29: 54%	
				30–34: 17%	
				≥34: 15%	
Headen et al. (2015)	US	6,489	Nationally representative sample with over sampling of Blacks, Hispanics, and low-income non-black, non-Hispanic populations	26.7 ± 5.1	White, Black, Hispanic
Hedderson, EP & Ferrara (2010)	US	1,134	General population	15–45	NHW, Hispanic, African American, Asian, Other
Herring et al. (2008)	US	94	General population	18–25: 73%25–42: 27%	Black/African American, Other
Hunt et al. (2013)	US	199,107	General Population	Underweight: NR	NHB, NHW
				Normal weight: 26.1	
				Overweight: 26.4	
				Obese: 26.7	
Kim et al. (2014)	US	660,038	General population	20–40+	Black, White, Hispanic, Asian/Pacific Islander
Kinnunen et al. (2016)	Norway	632	General population	Western European: 31.0 ± 4.4	Western European, South Asian, Middle Eastern, East Asian, Eastern European
				South Asian: 28.4 ± 4.3	
				Middle Eastern: 29.7 ± 5.5	
				East Asian: 31.0 ± 4.4	
				African: 28.5 ± 5.2	
				Eastern European: 28.7 ± 4.4	
Koleilat & Whaley (2013)	US	23,840	Hispanic women	<20: 13%	Hispanic - English Speaking, Hispanic - Spanish Speaking
				20–35: 74%	
				>35: 13%	
Kowal, Kuk & Tamim (2012)	Canada	6,233	General population	<20: 2%	Immigrant vs. non-immigrant, Aboriginal, British Isles/French, European, Other, North American
				20–29: 42%	
				30–39: 52%	
				≥40: 4%	
Krukowski et al. (2013)	US	4,619	General population	25.5 ± 6.79	Caucasian, African American, Hispanic
Larouche et al. (2010)	Canada	960	General Population	32 ± 4.8	Caucasian, Black, East Asian, West Asian/Arab, Latin American, South Asian
					Immigrant <5 years, immigrant 5–10 years, immigrant >10 years, non-immigrant
Leonard et al. (2017)	US	7,539	General population	26.9 ± 5.3	NHW/other, NHB, Hispanic
Lindberg et al. (2016)	US	7,385	General population	18–19: 2.7%	NHW, NHB, Hispanic, Other
				20–24: 12.3%	
				25-29: 30.8%	
				30–34: 36.2%	
				35–39: 15.1%	
				>40: 3.0%	
Magriples et al. (2013)	US	418	General population	20.7 ± 2.6	African American, Non-African American
Mendez et al. (2014)	US	55,608	General population	<20: 7%	NHB, NHW
				20–29: 46%	
				>35: 47%	
Mendez et al. (2016)	US	73,061	General population	<20: 7.5%	Black, White
				20–29: 45.5%	
				>30: 46.9%	
Pawlak et al. (2013)	US	230,698	General population	27.9 ± 6.1	NHW, Hispanic, NHB, Other
Shieh & Wu (2014)	US	56	Low income, predominately Black and Hispanic women	26.3 ± 6.3	Black, Hispanic
Sommer et al. (2014)	Norway	728	General population	29.4 ± 4.9	Europe, South Asia, Middle East, South/Central Africa, East Asia
Sridhar et al. (2014)	US	4,145	General population	Inadequate GWG: 33.4 ± 5.1	NHW, African American, Asian, Hispanic
				Adequate GWG: 33.5 ± 4.7	
				Excessive GWG: 32.5 ± 4.8	
Torloni et al. (2012)	US	1,762	High risk women	Majority 20–34	African American, Caucasian
Tovar et al. (2012)	US	952	Predominantly Hispanic women	22.7 ± 4.9	Three groups by score of acculturation (PAS score)
Walker, Cheng & Brown (2014)	US	250,857	General population	18–24: 38%	Two racial/ethnic groups: Hispanic and NHW further subdivided by border residency: NH-W-Border, NH-W-Non-border, Hispanic border, Hispanic Non-border
				≥25: 62%	
				Normal weight: 27.4 ± 4.5	
				Overweight: 28.4 ± 4.5	
				Obese: 28.4 ± 4.7	

**Notes.**

Abbreviations GDM gestational diabetes mellitus GWG gestational weight gain LGA large for gestation age NHW non-Hispanic White NHB non-Hispanic Black US United States PAS psychological acculturation scale

Ethnicities and races are reported as they were by the original authors.

A total of 46 studies (41 unique samples) did not compare GWG patterns using the 2009 IOM guidelines or the World Health Organization (WHO) BMI cut points and were subsequently described narratively. Of the 46 articles who did not use the 2009 guidelines, 43% used the 1990 IOM guidelines, 28% did not classify GWG into categories, 20% used arbitrary cut-offs, and 9% used other guidelines. Study characteristics can be found in Table S2. The remaining 40 studies that used the updated IOM guidelines were assessed quantitatively; 27 of which were included in the meta-analyses. Study characteristics that used the recent GWG guidelines can be found in Table 1.

The majority of included articles (87%) were from studies conducted in North America (largely the US). Studies were also performed in Europe (9%), Asia (2%) and Africa (2%). Sample sizes ranged from 56 (Shieh & Wu, 2014) to just over 600,000 (Kim et al., 2014) women and included women ranging in age from under 20 to over 40 years, with most women being within the ages of 20–29 years. Articles that were quantitatively analyzed most frequently included the racial/ethnic groups of White/Non-Hispanic White (72%), Black/Non-Hispanic Black (66%), Hispanic (45%), and Asian (20%). Over half of the studies (62%) reported on nationality. Language (15%) and acculturation (15%) were the least reported indicators of culture. Sixteen studies included in the meta-analysis reported pre-pregnancy BMI by race. In 13 of these studies, Black participants had a higher pre-pregnancy BMI or were more likely to be overweight or obese than their White, Hispanic and Asian counterparts. More detailed descriptions of pre-pregnancy BMI by race from studies included in the meta-analysis can be found in Table S3. Our main outcome, GWG, was most often calculated with the use of self-reported pre-pregnancy BMI. An overview of how GWG was determined in each study can be found in Table S4.

**Table 2:** Main findings from articles using the 2009 IOM guidelines listed in alphabetical order, by author.

**First author, year**	**Main outcome**	**Summary of GWG results**
Badreldin, Grobman & Pool (2018)^a^	GWG	• Hispanic and Black women were more likely to experience weight loss or guideline discordant GWG
Berggren, Stuebe & Boggess (2015)^a^	LGA	• Caucasian: 32.5% below and 43.6% above guidelines • African American: 22.6% below and 66% above guidelines • Hispanic: 33.4% below and 36.1% above guidelines • Did not comment on if this was significant; main outcomes were LGA and GDM
Bodnar et al. (2001)^a^	Adverse birth outcomes	• White: 17.2% below and 50.1% above • Black: 25% below and 47.3% above • Black women gained less weight than White women
Bogaerts et al. (2012)	Pre-pregnancy BMI and GWG	• Dutch and Turkish, in comparison to Belgian or Moroccan, were independently associated with EGWG
Bowers et al. (2013)^a^	Excess fetal growth	• Asian ethnicity, in comparison to NHW, NHB and Hispanics, was positively associated with IGWG
Cavicchia et al. (2014)^a^	GDM	• White women exceeded guidelines the most; Hispanic women the least • NHW positively associated with excessive GWG
Chaffee et al. (2015)^a^	Excessive GWG and association with mid-life obesity	• Non-black Hispanic had higher prevalence of EGWG vs. Non-black-non-Hispanic and NHB
Chang et al. (2017)^a^	Rick factors for discordant GWG	• Hispanic women had lower risk of EGWG than non-Hispanic women
Chasan-Taber et al. (2016)	GWG and pre-pregnancy BMI	• US born women were more likely to gain excessively than those born in Puerto Rico or the Dominican Republic. • No significant difference in GWG by acculturation
Cheng et al. (2015)^a^	Discordant GWG and perinatal outcomes	• Asian women had higher risk of inadequate GWG than NHW • No difference between Asian subgroups (when confounders were accounted for)
Chihara et al. (2014)^a^	Birth weight	• Pacific Islander and Hawaiian, in comparison to White and Asian women, had the highest prevalence of EGWG
Cohen et al. (2016)^a^	GWG	• Black women without high school education were less likely to have EGWG than those with a high school education • White women without high school education were more likely to have EGWG than those with a high school education • Education was not associated with IGWG. This relationship was not modified by race-ethnicity
Cox Bauer et al. (2016)^a^	Validity of IOM guidelines for women with OB	• Suggested greater prevalence of weight gain in White women
Deputy et al. (2015)	Adherence to 2009 IOM guidelines	• Among normal weight women, NHB, Asian and Hispanics were positively associated with IGWG • Women who were OW, NHB or Alaskan native were positively associated with IGWG
Fontaine et al. (2012)^a^	GWG	• Black women were significantly more likely to enter pregnancy OB (34% vs. 24%), but White women gained more weight than Blacks in all BMI categories
Rothberg et al. (2011)	GWG & post-partum weight retention	• NHW more likely to exceed guidelines in all BMI categories, NHB women had similar trajectory • Hispanic women had most favourable outcomes
Haile et al. (2017)^a^	Delayed onset of lactation	• NHW women had the highest prevalence of EGWG
Harris et al. (2015)^a^	Adherence to 2009 IOM Guidelines	• Race/ethnicity was not significantly associated with meeting the IOM guidelines
Headen et al. (2015)^a^	GWG	• Black and Hispanic women positively associated with inadequate GWG in comparison to White women when BMI <25 • No interaction between race & EGWG
Hedderson, EP & Ferrara (2010)	GWG and risk of GDM	• Association between race and rate of weight gain (up until GDM screening) was borderline significant (races not reported)
Herring et al. (2008)^a^	Modifiable mid-pregnancy behaviours & excessive GWG	• Race/ethnicity did not influence GWG
Hunt et al. (2013)^a^	Birth weight	• NHB: 35% below and 41% above guidelines • NHW: 24% below and 49% above guidelines • Did not comment on if this was significant (since birth weight was main outcome)
Kim et al. (2014)^a^	LGA	• Black: 22% below and 49% above guidelines • White: 15% below and 53% above guidelines • Hispanic: 18% below and 50% above guidelines • Asian/Pacific Island: 23% below and 36% excessive • No comment on whether this result was significant; LGA was main outcome
Kinnunen et al. (2016)	GWG	• Eastern European women gained significantly more weight than Western European, South Asian, Middle Eastern, Africa and East Asian women.
Koleilat & Whaley (2013)	EGWG	• Hispanic English speaking women more likely to exceed guidelines than Hispanic Spanish speaking women • Women that preferred Spanish were 42% less likely to exceed guidelines
Kowal, Kuk & Tamim (2012)	GWG	• Immigrants to Canada gained less weight and were 1.5 times more likely to gain below guidelines vs. non-immigrant Canadian women
Krukowski et al. (2013)^a^	EGWG	• Lower odds of exceeding guidelines if African American or Hispanic
Larouche et al. (2010)^a^	EGWG	• Latin American women gained more weight than South Asian women
Leonard et al. (2017)^a^	High birthweight and childhood overweight/obesity	• Significant relationship between EGWG and overweight in late childhood in NHW women • Overnutrition in pregnancy independently affects child body composition in child development in NHW women
Lindberg et al. (2016)	Prevalence of discordant GWG	• NHW was identified as a risk factor for EGWG • NHB was identified as a risk factor for IGWG
Magriples et al. (2013)^a^	Blood pressure changes	• African American women had less GWG than their non-African American counterparts (Latina, White or ‘other’ race)
Mendez et al. (2014)^a^	GWG or loss	• Black women more likely to be OW/OB prior to pregnancy • Black women also had IGWG or had weight loss in comparison to their White counterparts
Mendez et al. (2016)^a^	Relationship between GWG, pre-pregnancy BMI and hypertension disorder	• Black women were more likely to have IGWG compared to White women
Pawlak et al. (2013)^a^	GWG	• Hispanic women had increased risk of inadequate gain and decreased risk of EGWG in comparison to NHW • Black women had an increased risk of inadequate gain in comparison to NHW • New immigrants to US (<9 years) had increased risk of IGWG compared to US-born women
Shieh & Wu (2014)^a^	Relationship between OB, GWG and Depressive symptoms	• Black women more likely to exceed guidelines than Hispanic women
Sommer et al. (2014)	Changes in adiposity & association to GDM	• South and Central African women gained less total fat mass and truncal fat than European, South Asian, Middle Eastern, and East Asian women. • No significant differences present in discordant or concordant GWG across all racial/ethnic groups.
Sridhar et al. (2014)^a^	Association between GWG and offspring OW/OB at age 2–5 years	• White women were more likely to exceed guidelines; Asian or Black women more likely to fall below
Torloni et al. (2012)^a^	BMI and its relation to preterm birth, and if ethnicity is an associated risk	• Black: 20% below and 65% above guidelines • White: 22% below and 66% above guidelines • Did not comment on if this was significant; PTB was main outcome
Tovar et al. (2012)	Acculturation and GWG	• Women born in US had greater average GWG than women born in Puerto Rico/Dominican Republic
Walker, Cheng & Brown (2014)	Birth outcomes	• Hispanic women, in comparison to NHW, had a lower risk of inadequate GWG and decreased risk of EGWG • Border residency did not impact GWG

**Notes.**

a Data used in meta-analyses.

Abbreviations LGA large for gestational age GDM gestational diabetes mellitus BMI body mass index GWG gestational weight gain EGWG excessive gestational weight gain NHW non-Hispanic White NHB Non-Hispanic Black IGWG inadequate gestational weight gain IOM Institute of Medicine OB obese OW overweight US United States PTB Pre-term birth

### Primary outcomes

Most articles (82%) that used the 2009 IOM guidelines reported that GWG differed by race/ethnicity. Table 2 provides an overview of these findings. Commonly performed comparisons were between White, Black, Hispanic and Asian women with a minority of studies examining women of other ethnic/racial background or by acculturation status. Collectively, there were differences in discordant GWG patterns across cultural groups.

#### Excessive GWG

**Figure 2:**
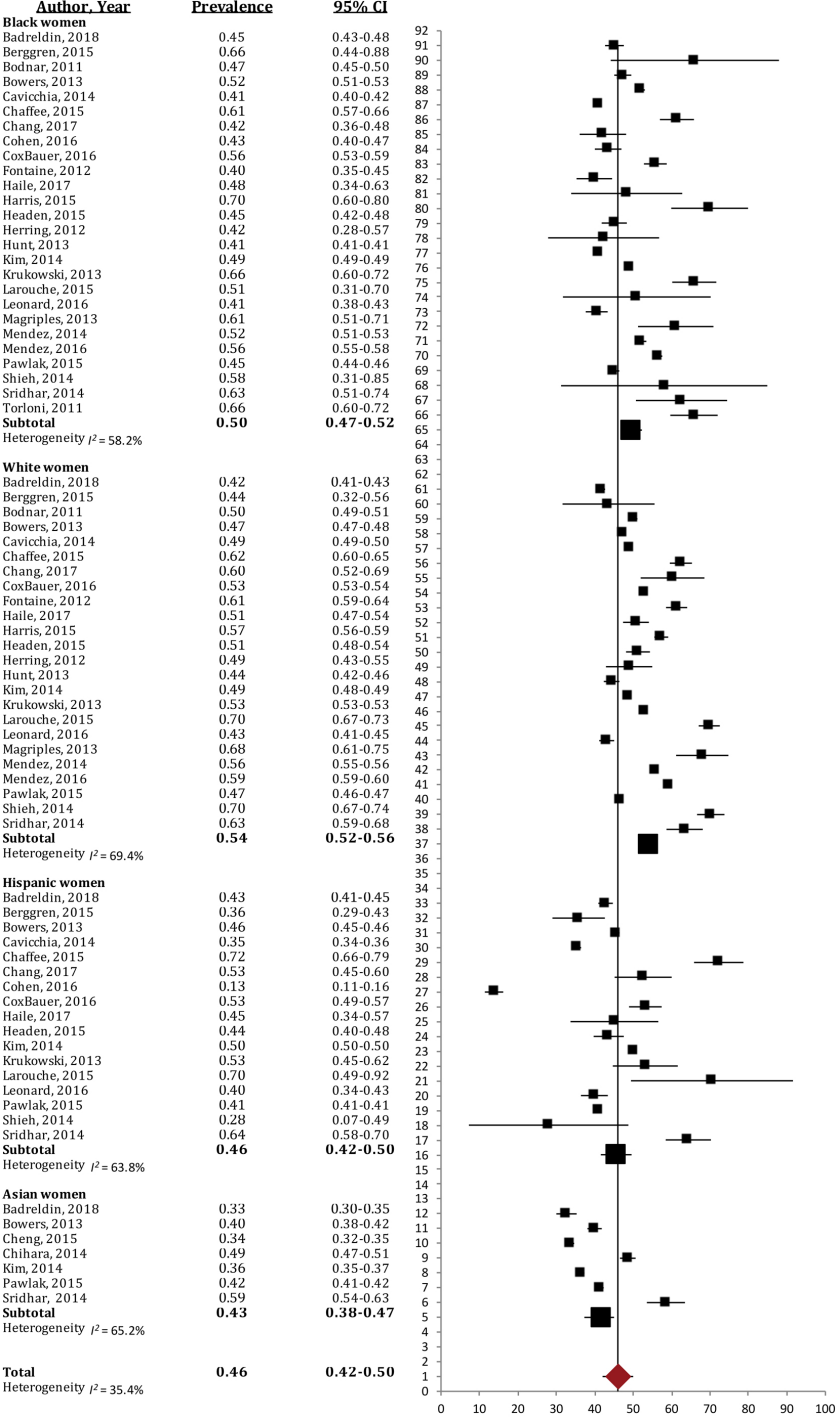
Forest plot of the prevalence of excessive gestational weight gain in Black, White, Asian and Hispanic women. The prevalence of women exceeding the 2009 IOM gestational weight gain guidelines, broken down by racial/ethnic groups. Large black sqaure represents the weighted prevalence in each group.

Overall, almost half of the women of White, Black, Hispanic and Asian racial/ethnic groups gained in excess of the current IOM guidelines (46%, 95% CI [42%–50%]; *I*^2^ = 35.4%; Fig. 2). White women experienced excessive GWG most often (54%, 95% CI [52%–56%]; *I*^2^ = 69.4%), and significantly more so than Asian (43%, 95% CI [38%–47%]; *I*^2^ = 65.2%) and Hispanic women (46%, 95% CI [42%–50%]; *I*^2^ = 63.8%); Black women had higher prevalence of excessive GWG (50%, 95% CI [47%–52%]; *I*^2^ = 58.2%) than their Hispanic and Asian counterparts; however, this difference was not significant.

#### Inadequate GWG

**Figure 3:**
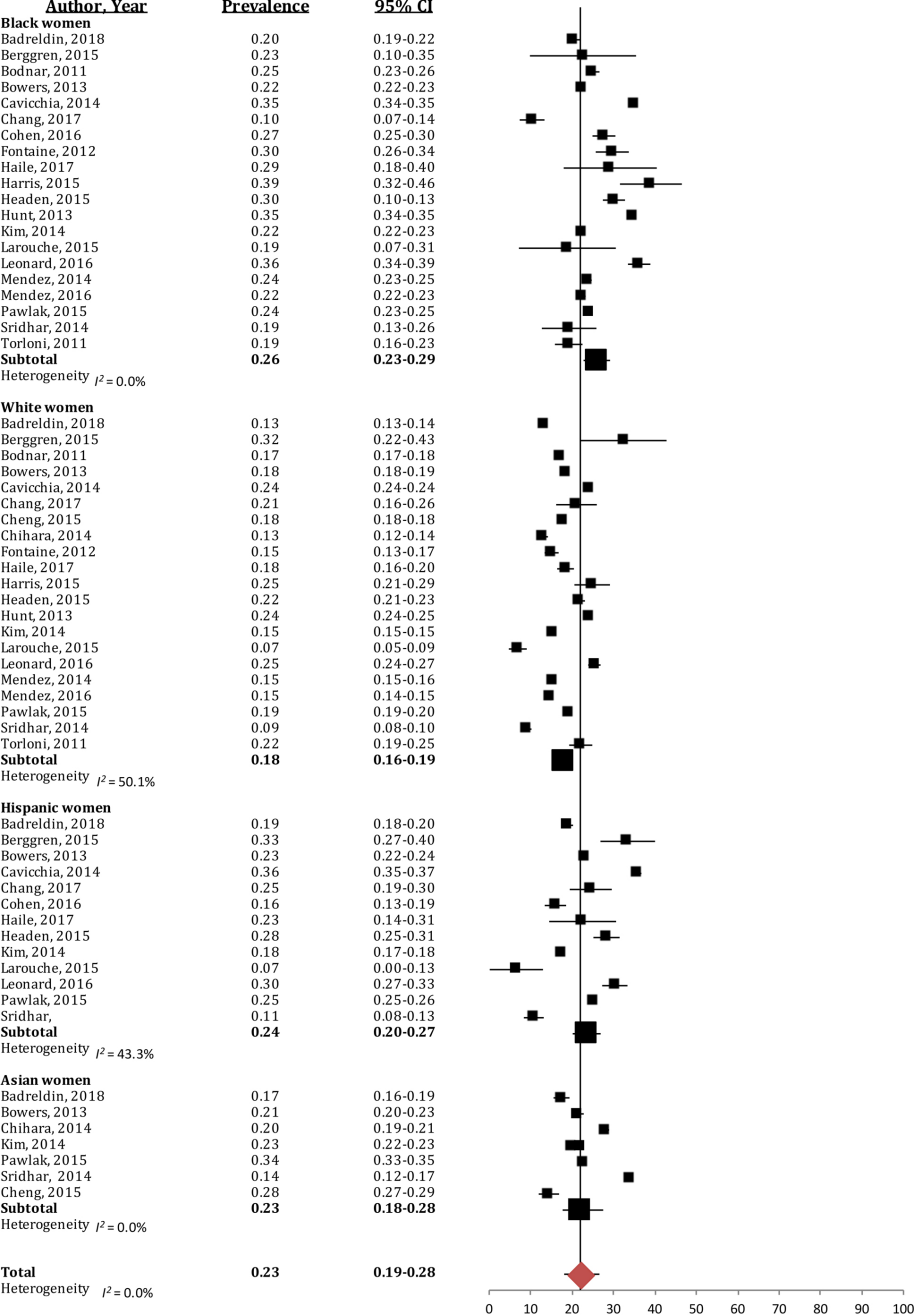
Forest plot of the prevalence of inadequate gestational weight gain in Black, White, Hispanic and Asian women. The prevalence of women gaining below the 2009 IOM gestational weight gain guidelines, broken down by racial/ethnic groups. Large black sqaure represents the weighted prevalence in each group.

Inadequate GWG was much less prevalent, with a quarter of women not meeting the guidelines (23% (95% CI [19%–28%]); *I*^2^ = 0%; Fig. 3). Black women had the highest prevalence of inadequate GWG (26% (95% CI [23%–29%]); *I*^2^ = 0%), which was significantly greater than the prevalence in White women (18% (95% CI [16%–19%]); *I*^2^ = 50.1%). Hispanic and Asian women presented with similar prevalence of inadequate GWG (Hispanic: 24% (95% CI [20%–27%]); *I*^2^ = 43.3%, Asian: 23% (95% CI [18%–28%]); *I*^2^ = 0%), and this was not significantly different than other racial/ethnic groups.

#### GWG Differences due to Acculturation

Four studies used immigration status as a way of classifying acculturation. Three studies (Kowal, Kuk & Tamim, 2012; Tovar et al., 2012; Chasan-Taber et al., 2016) showed that immigrant women were at a higher risk of discordant GWG with respect to the current IOM guidelines, while one (Larouche et al., 2010) found no significant difference. Kowal et al. found that women who immigrated to Canada were 1.5 times more likely to gain below the IOM recommendations; (Kowal, Kuk & Tamim, 2012) in contrast with previous findings from Larouche et al. who illustrated that immigration status did not impact GWG patterns in Canadian immigrants (Larouche et al., 2010). The latter study, which was restricted to births delivered at a local metropolitan hospital, initially found an association between immigration status and GWG, yet this association was no longer significant when hypertension was added as a covariate. When using an acculturation score, Chasan-Taber et al. (2016) found no association between acculturation status and adherence to IOM guidelines. However, they did report that women born in the continental U.S were more likely to gain excessively than those born in Puerto Rico or the Dominican Republic (Chasan-Taber et al., 2016). Similarly, Tovar et al. reported that Puerto Rican women whose families had been in the US longer were at a higher risk of excessive GWG than newer Puerto Rican immigrants (Tovar et al., 2012). Koleilat & Whaley (2013) characterized acculturation by preferred language of US-residing Hispanic women and showed that women who preferred to speak Spanish were 42% less likely to exceed the IOM guidelines than those who preferred the English language.

### Secondary outcomes

Seven papers included stratification of maternal and fetal outcomes by race/ethnicity. White women were more likely than Hispanic women to have an unplanned caesarean section (Walker, Cheng & Brown, 2014); this relationship was opposite when comparing White and Asian women (Cheng et al., 2015). Bodnar et al. (2001) reported similar rates of caesarean section between Black and White populations. Disparities in caesarean section rates have also been linked to the location of residence; Hispanic-American women residing near the US-Mexico border had a higher risk of caesarean section than Hispanic women living away from the border (Walker, Cheng & Brown, 2014).

Three studies reported similar prevalence rates of GDM among Black and White women (Bowers et al., 2013; Cavicchia et al., 2014; Kim et al., 2014). Two studies included Asian ethnicities and illustrated a greater prevalence of GDM in this ethnic group in comparison to White women, (Cheng et al., 2015) or to White, Black and Hispanic women (Kim et al., 2014). Similarly, minority groups were less likely to have pregnancy-induced hypertension, where Hispanic women experienced hypertension less than their White and Black counterparts, (Cavicchia et al., 2014) and Asian women less than women of Caucasian descent (Cheng et al., 2015). White women have been shown to be at a higher risk for delivering a macrosomic or LGA infant when compared to Black, Asian and Hispanic groups (Bodnar et al., 2001; Walker, Cheng & Brown, 2014; Cheng et al., 2015).

When stratifying these outcomes by immigration patterns, a Canadian study reported similar caesarean section rates among immigrants to Canada regardless of time of arrival. However, non-immigrants had a lower percentage of caesarean rates than all immigrants, irrespective of status (Larouche et al., 2010).

### Risk of bias

A summary of the risk of bias assessments is presented in Table S5. All studies had a low overall risk of bias; most biases originated from the use of convenience sampling (selection bias), or from self-reported pre-pregnancy weight (detection bias).

### Publication bias

No publication bias was present for studies examining excessive gestational weight gain in all four racial/ethnic groups (*p* > 0.05). There was evidence of publication bias in the studies that addressed inadequate GWG in the Asian population (*p* = 0.035), but not in the other racial/ethnic groups (*p* > 0.05). The bias in the Asian population is most likely driven by the low sample size included in the published studies, and the limited published literature focusing on the Asian population outside of the US that met our meta-analysis eligibility criteria.

### Subgroup analyses

Substantial heterogeneity (*I*^2^ > 50%) was present for all racial/ethnic groups for excessive GWG. When sufficient data were available, subgroup analyses were conducted within each racial/ethnic group to explore potential causes of heterogeneity. The results of the subgroup analysis indicated no significant differences (±5%) between studies for the region of study (US vs. other) or population (healthy vs. women classified as high risk). As expected, the subgroup of studies with high selection bias had a greater effect size and lower heterogeneity in the White, Black and Hispanic ethnic groups; there were not sufficient numbers to assess this outcome in the Asian subgroup. High selection bias was driven by single-site participant recruitment, most likely leading to a more homogenous group, which could explain the improved outcomes. No differences were found when comparing study design (prospective vs. retrospective) in White, Black and Hispanic groups. However, removal of one study with a prospective cohort design in the Asian group reduced *I*^2^ from 65.1 to 36.6. Sample size did not impact our results within the White, Hispanic and Asian racial/ethnic groups; whereas studies with small sample sizes (*N* < 1, 000) reduced the level of heterogeneity in the Black group (*I*^2^ = 58.1 vs. 11.5). No differences were found when comparing subgroups for single vs. multi-site studies for White, Hispanic and Asian groups; however, when looking at single sites alone within studies that assess excessive GWG in Black women, heterogeneity was lower than in studies with multi-sites (*I*^2^ = 58.1 vs. 35.2).

### Narrative synthesis of studies using ‘other’ guidelines

The findings from the subset of 46 studies that did not use the 2009 IOM GWG guidelines are summarized in Table S6. The findings among these studies were remarkably similar to those applying the recent IOM guidelines, whereby there was a trend for increasing GWG gain from Asian, Hispanic, Black and White women, with Asian women gaining the least and White women gaining the most (Ademowore, Courey & Kime, 1972; Keppel & Taffel, 1993; Caulfield, Witter & Stoltzfus, 1996; Caulfield, Stoltzfus & Witter, 1998; Hickey et al., 1997; Schieve, Cogswell & Scanlon, 1998; Hardy, 1999; Taffel, Keppel & Jones, 2003; Rosenberg et al., 2005; Stotland et al., 2006; Wells et al., 2006; Ochsenbein-Kollble et al., 2007; Ellerbe et al., 2013; Bentley-Lewis et al., 2014; Sackoff & Yunzal-Butler, 2014). Interestingly, few studies reported minority groups experiencing greater discordant GWG than White women. Studies examining acculturation either through language or immigration status, in the narrative subset, also demonstrated similar results to those seen with papers utilizing the 2009 IOM guidelines.

Acculturation status influenced adherence to the older 1990 guidelines. Hackley et al. reported that in US residents, a higher proportion of Spanish-speaking Hispanic women had weight gain concordant with IOM recommendations; yet, language preference was not significantly associated with adherence to these guidelines (Hackley et al., 2010). Immigration status appeared to play a role in GWG trajectories, wherein Mexican-born US residents were more likely to have inadequate GWG in comparison to their US-born counterparts (Heilemann et al., 2000). This relationship was further highlighted by Chasan-Taber et al. (2008), who reported that residing in the US for under ten years resulted in a lower risk of exceeding guidelines compared to third generation women. Similarly, when looking at pregnant women of Mexican origin, US-born women have higher rates of excessive GWG than foreign-born women (Sparks, 2009), indicating the role acculturation may play in achieving the IOM guidelines.

Numerous studies explored more diverse racial/ethnic groups (Neser, 1963; Allen et al., 1994; Frisbie, Forbes & Hummer, 1998; Larouche et al., 2010; Bogaerts et al., 2012; Kowal, Kuk & Tamim, 2012; Gaillard et al., 2013; Hernandez-Rivas et al., 2013; Sommer et al., 2014; Bahadoer et al., 2015; Deputy et al., 2015; Kinnunen et al., 2016) but there were too few similarities within the data to compare GWG patterns. These studies are included in the summaries presented in Table 2 (2009 IOM guidelines).

A sub-set of studies whose data were colleted prior to the inception of the evidence-based 2009 GWG guidelines reported no association between culture and discordant GWG (Hickey et al., 1990; Hickey et al., 1993; Hickey et al., 1995a; Hickey et al., 1995b; Hickey et al., 1996; Petitti, Croughan-Minihane & Hiatt, 1991; Allen et al., 1994; Walker & Kim, 2002; Savitz et al., 2011; Hernandez-Rivas et al., 2013; Sackoff & Yunzal-Butler, 2014; Widen et al., 2015); however, few recent studies have shown this to be true (Cheng et al., 2015; Harris et al., 2015).

## Discussion

This is the first systematic review to critically analyze the differences in GWG across different ethnic and cultural groups. During the 2009 update of the IOM guidelines, culture was recognized as a potential moderator for achieving appropriate weight gain. Due to the lack of empirical evidence at that time, it was noted that the magnitude of culture’s influence remained unknown. As such, the present review provides much-needed insight related to the role of culture and achieving a healthy pregnancy. Regardless of which set of guidelines were used, a high proportion of studies (77%) reported some degree of cultural influence—whether that be race, ethnicity, language or immigration status—on achieving optimal GWG. Our findings show that White women were more likely to exceed the IOM guidelines than their Asian and Hispanic counterparts, but White and Black women had a similar prevalence of exceeding these guidelines. Women of a minority group were generally at an increased risk of inadequate GWG and a decreased risk of adequate GWG compared to White women. Thus, Black women have the highest risk of overall discordant GWG since they often tend to under-gain *and* over-gain. When looking at immigrant vs. non-immigrant populations, the former is more at risk of inadequate GWG. Not surprisingly, only 25% of women gained weight concordant with the current recommendations.

Interestingly, there were a greater number of studies that reported Black women gaining more weight on average, and exceeding the 1990 IOM guidelines than White women, compared to the updated guidelines where Black and White women had a similar prevalence of excessive GWG. While improvements in socioeconomic discrepancies (Firebaugh & Farrell, 2016) may explain similarities between excessive GWG in recent years, the reasons behind why Black women are more at risk for inadequate GWG are still largely misunderstood. Possible contributing factors could be lower educational attainment (Cohen et al., 2016; Ryan & Bauman, 2016), high psychosocial stress (Headen et al., 2015) or differences in prenatal health counselling (Whitaker et al., 2016).

Hispanic women were most often reported to gain below current IOM guidelines, and are at the least risk of exceeding them. Hispanic women may be at a higher risk for inadequate GWG due to perceived discrimination by their health care providers during prenatal care, thus limiting discussions about healthy pregnancy weight gain (Attanasio & Kozhimannil, 2015). Furthermore, the recent immigration status of many Hispanic Americans may also explain the lower GWG, since recent immigrant women may not have adopted the social and cultural customs shared in North America. In support of this, generational studies have shown that second-generation Hispanic immigrants have higher rates of smoking and drinking, (Guendelman & English, 1995) and adopt poorer nutritional food habits (Akresh, 2007) compared to first-generation immigrants.

Similar to the other racial/ethnic groups, excessive GWG was common among Asian women, albeit, they had the lowest prevalence of all groups. Evidence indicates that Asian populations have a higher percentage of abdominal body fat at a lower BMI, increasing the risk of cardio-metabolic diseases (Barba et al., 2004). This leads to the questioning of the efficacy of IOM-based GWG recommendations within this population. Eu et al. reported discrepancies between the IOM guidelines and optimal GWG in Asian populations whereby the recommended optimal range for women in Asia was wider for each category with greater acceptance of lower GWG, namely weight loss in obese women (Ee et al., 2014). It is important to consider that, over time, Asians who immigrate to the US begin to adopt lifestyle behaviors leading to weight gain patterns typically observed in the Caucasian-American population (Lauderdale & Rathouz, 2000). Using the WHO BMI and IOM GWG guidelines may misclassify pregnancy risks in Asian women whose genetic profile differs from Caucasian populations. Thus, anthropometric differences and the potential for misclassification further highlights the need for culturally diverse guidelines.

There was not one racial/ethnic group that was protected from adverse maternal-fetal outcomes. However, patterns were present in the prevalence of poor outcomes between groups. For example, White women were more at risk for adverse outcomes such as unplanned cesarean sections and having a macrosomic or LGA infant. Epidemiological data has shown that both outcomes are more prevalent in women who gain in excess of the IOM GWG guidelines (Stotland, Hopkins & Caughey, 2004; Ferraro et al., 2012). In contrast, we found a higher risk for GDM among Asian women. A recent study that looks at prevalence and risk factors associated with GDM supports this finding (Pu et al., 2015). Pu and colleagues looked at the relative contributions to GDM risk and reported a significant interaction between race, specifically Asian, and family history of type 2 diabetes. Additionally, foreign-born status also increased the risk of GDM. Since the studies included in our meta-analysis were conducted in the US, this could help explain the findings.

Culture plays a role in beliefs and perceptions. For example, culture plays an influential role in dietary patterns during pregnancy, with the majority of women reporting eating foods that are culturally encouraged (Brooten et al., 2012; Guelfi et al., 2015). Food habits and beliefs during pregnancy, such as eating for two or restricting certain foods are often transferred from generation to generation (Carruth & Skinner, 1991). Moreover, perceptions about GWG also vary by cultural beliefs and are often linked to how cultures view body size (Groth, Morrison-Beedy & Meng, 2012). Black women perceive themselves to be of normal weight at higher BMI categories than White women (Bennett & Wolin, 2006). Similarly, Hispanic women express concern of gaining weight excessively over pregnancy, but still define attractiveness as having a fuller body and hips (Tovar et al., 2010). While beliefs vary between broader racial groups, varied perceptions also exist between smaller ethnic subsets. For example, African American women perceived a greater GWG as healthy compared to Caribbean Black women who regard lower GWG as healthy (Brooten et al., 2012).

This is the first systematic review to critically analyze the differences in GWG across different ethnic and cultural groups. A large number of papers and thus sample size allowed for a thorough review of the evidence. Moreover, the inclusion of the 1990 guidelines allowed for an in-depth analysis of trends in GWG and shifting cultural definitions. This study has several limitations. While we sought a globally representative sample, 87% of the articles meeting inclusion were carried out in North America (especially the US), most of which compared a small number of racial/ethnic groups (Black, White, Hispanic and Asian). As such, this limits the generalizability of our results to other cultural subgroups and the strength of recommendations made by the IOM on a universal scale. Moreover, the limited literature present on cultural differences in secondary outcomes did not provide clear trends of which groups are more at risk of pregnancy-related complications than others. Overall, there is not one racial/ethnic subgroup with a significantly lower risk profile regarding maternal-fetal health outcomes. Nevertheless, immigrants to North America tended to have better odds of achieving optimal health in comparison to non-immigrants.

Some bias was present in the studies included in this analysis. High selection bias swayed the results in the Hispanic ethnic group in a way such that women gained less weight than in studies with low bias. Studies that had high selection bias often recruited participants or abstracted data from charts from one study setting, thus potentially favoring the women with more optimal health. Our outcome, GWG, was often based on self-reported data. While there is some concern around the accuracy of self-report data, Hinkle et al. have shown that a mother’s recall of her GWG may be an appropriate substitute for missing data from a birth certificate, within one year postpartum (Hinkle et al., 2013).

Lastly, even with the inclusion of a variety of factors, it is difficult to quantify the values and beliefs that make up an individual’s culture. Perhaps the most difficult aspect of culture is that, with increasing globalization, cultures are constantly evolving, giving way to a more universal, common set of beliefs. Further, the commonly used classifications for race/ethnicity are quite broad and ignore the differences between subgroups; (Kumanyika & Krebs-Smith, 2001) classifying a group as simply “White” or “Black” can accommodate an extremely diverse cohort of people and subsequently beliefs and behavior patterns. Despite this, the current systematic review summarizes the available evidence that pertains to known constructs of culture and does demonstrate the importance of continued work in this area.

## Conclusion

In summary, this systematic review synthesized data from 86 articles looking at cultural differences in achieving the IOM GWG guidelines, with 40 of the articles comparing to the current evidence-based recommendations. The vast majority of women experienced discordant GWG, and this was consistently shown to be culturally dependent, wherein minority groups such as Black, Hispanic and Asian women are more likely to gain below current recommendations, and White women to exceed them. Studies examining Black women indicated they were at risk of both inadequate and excessive GWG. Less acculturated women (mainly to the US), are at a greater risk of inadequate GWG. Future research should continue to address this topic, and place a special focus on acculturation due to the increasing migration and cultural globalization in today’s society. The data presented here suggest that culture should be considered in future policy decisions. In a practical setting, care should be taken to understand and be mindful of individual needs when discussing prenatal behaviors to achieve optimal GWG.

##  Supplemental Information

10.7717/peerj.5407/supp-1 Supplemental Information 1 PRISMA flow diagram

10.7717/peerj.5407/supp-2 Supplemental Information 2 PRISMA checklist

10.7717/peerj.5407/supp-3 Supplemental Information 3 Supplemental Information

## Additional Information and Declarations

### Data Deposition

The following information was supplied regarding data availability: The research in this article did not generate any raw data or code. This article is a systematic review and meta-analysis. All findings are reported in the text.
