# Supplementary material for: Addressing cultural, racial and ethnic discrepancies in guideline discordant gestational weight gain: a systematic review and meta-analysis
Source: PeerJ. 2018 Aug 27;6:e5407. doi: 10.7717/peerj.5407 (PMC6118200; doi:10.7717/peerj.5407)
Supplement: Supplemental Information 1 [file peerj-06-5407-s001.docx]

Update April 2018

N = 528

Update May 2017

N = 380

Update July 2016

N = 150

Initial

N = 3,628

**Studies included in systematic review** N = 86

Full-text article assessed for eligibility (independently reviewed in duplicate)

N = 313

**Articles excluded with reason**

N= 226

- Outcomes n *=* 96
- Exposure n = 46
- Conference Proceedings n = 22
- Population n = 4
- Duplicate n = 33
- Study Design n = 24
- Comparator n = 1

Studies excluded based on review of titles and abstract N= 3,527

Initial screening of unique title and abstracts N = 3,840

Meta-analysis

N = 27

Used 2009 Guidelines

N = 40

Used Alternative Guidelines

N = 46

Studies identified through database searching

N = 4,686

Duplicate publications N = 1,788
