# Supplementary material for: Addressing cultural, racial and ethnic discrepancies in guideline discordant gestational weight gain: a systematic review and meta-analysis
Source: PeerJ. 2018 Aug 27;6:e5407. doi: 10.7717/peerj.5407 (PMC6118200; doi:10.7717/peerj.5407)
Supplement: Supplemental Information 2 [file peerj-06-5407-s002.doc]

| **Section/topic** | **#** | **Checklist item** | **Reported on page #** |
| --- | --- | --- | --- |
| **TITLE** | | |  |
| Title | 1 | Identify the report as a systematic review, meta-analysis, or both. | 1 |
| **ABSTRACT** | | |  |
| Structured summary | 2 | Provide a structured summary including, as applicable: background; objectives; data sources; study eligibility criteria, participants, and interventions; study appraisal and synthesis methods; results; limitations; conclusions and implications of key findings; systematic review registration number. | 3 |
| **INTRODUCTION** | | |  |
| Rationale | 3 | Describe the rationale for the review in the context of what is already known. | 4  “A previous review by Headen *et al.* examined the associations between racial/ethnic identities and GWG (Heade*n et a*l., 2012). This narrative was limited in its inclusion criteria, with a focus on White, Black and/or Hispanic women within the United States (U.S.), it only compared GWG to the 1990 IOM guidelines, and excluded pregnancies complicated by adverse maternal-fetal health outcomes. The conclusion of their review identified that greater research surrounding the social context of race and GWG was needed (Heade*n et a*l., 2012).” |
| Objectives | 4 | Provide an explicit statement of questions being addressed with reference to participants, interventions, comparisons, outcomes, and study design (PICOS). | 5-6  Under headings found on lines 106-125 |
| **METHODS** | | |  |
| Protocol and registration | 5 | Indicate if a review protocol exists, if and where it can be accessed (e.g., Web address), and, if available, provide registration information including registration number. | 5  “This systematic review was prospectively registered on the PROSPERO database (#CRD42015023399) and the protocol has been published elsewhere (Manyang*a et a*l., 2015).” |
| Eligibility criteria | 6 | Specify study characteristics (e.g., PICOS, length of follow-up) and report characteristics (e.g., years considered, language, publication status) used as criteria for eligibility, giving rationale. | 5-6  “This review systematically identified prospective and retrospective observational and cohort studies. The language of publication was not an exclusion factor and relevant translation was procured as necessary (NA and DFdS).” |
| Information sources | 7 | Describe all information sources (e.g., databases with dates of coverage, contact with study authors to identify additional studies) in the search and date last searched. | 4  “Ten bibliographic databases were searched including: Ovid MEDLINE; EMBASE; Clinicaltrials.gov; Cochrane Central Register of Controlled Trials; CINAHL; PsycINFO; Sociological Abstracts; Literature Latino-Americana e do Caribe em Ciencias da Saude (LILACS), IBECS; and, Cuba Medicina (CUMED).” |
| Search | 8 | Present full electronic search strategy for at least one database, including any limits used, such that it could be repeated. | Table S1 |
| Study selection | 9 | State the process for selecting studies (i.e., screening, eligibility, included in systematic review, and, if applicable, included in the meta-analysis). | 6  “Studies were included if they compared at least two different cultural groups. When a study looked at the outcome in only one cultural population, it was excluded.”  8  “When five or more studies were available to describe the rates of GWG under a specific racial/ethnic group, a meta-analysis was conducted.” |
| Data collection process | 10 | Describe method of data extraction from reports (e.g., piloted forms, independently, in duplicate) and any processes for obtaining and confirming data from investigators. | 7  “The results of the search were imported into Covidence (Cochrane, Melbourne, Australia), and then duplicates were removed prior to initial screening. Two independent reviewers (original search: TM and DfdS, update: NA and KD) screened the titles and abstracts of the search results and marked each as ‘include’, ‘exclude’ or ‘unsure’ based on the eligibility criteria. The full texts of the studies classified as ‘unsure’ or ‘include’ were then reviewed by the same two reviewers based on each of the eligibility criteria. Conflicts were resolved through consensus and discussion with a third reviewer (ZMF).” |
| Data items | 11 | List and define all variables for which data were sought (e.g., PICOS, funding sources) and any assumptions and simplifications made. | 6  “The primary outcome was inadequate or excessive GWG (hereafter referred to as discordant GWG), as defined by the IOM. Studies that used the 1990 guidelines were included but analyzed separately from studies that compared GWG to the updated 2009 guidelines. Secondary outcomes include maternal-fetal health outcomes such as large-for-gestational age (LGA), macrosomia, gestational diabetes mellitus (GDM), and hypertension.” |
| Risk of bias in individual studies | 12 | Describe methods used for assessing risk of bias of individual studies (including specification of whether this was done at the study or outcome level), and how this information is to be used in any data synthesis. | 9  “A modified Cochrane Risk of Bias Tool was used to assess the level of bias in each study included in the quantitative analysis. The Tool was modified for use in the evaluation of bias in prospective and retrospective study designs (Poitra*s et a*l., 2016).” |
| Summary measures | 13 | State the principal summary measures (e.g., risk ratio, difference in means). | 8  “Meta-analyses were completed to compare the proportion (and 95% confidence intervals [CIs]) of women in each study who experienced excessive or inadequate GWG within a racial/ethnic group. A random effects meta-analysis was conducted to provide an overall measure of effect (proportion with excessive/inadequate GWG) and 95% CIs for each population group (i.e. White, Black, Hispanic, Asian).” |
| Synthesis of results | 14 | Describe the methods of handling data and combining results of studies, if done, including measures of consistency (e.g., I^2^) for each meta-analysis. | 8  “Cochrane’s Q statistic and the *I^2^* statistic were used to assess heterogeneity between studies.”” |

Page 1 of 2

| **Section/topic** | **#** | **Checklist item** | **Reported on page #** |
| --- | --- | --- | --- |
| Risk of bias across studies | 15 | Specify any assessment of risk of bias that may affect the cumulative evidence (e.g., publication bias, selective reporting within studies). | 9  “A modified Cochrane Risk of Bias Tool was used to assess the level of bias in each study included in the quantitative analysis. The Tool was modified for use in the evaluation of bias in prospective and retrospective study designs (Poitra*s et a*l., 2016).” |
| Additional analyses | 16 | Describe methods of additional analyses (e.g., sensitivity or subgroup analyses, meta-regression), if done, indicating which were pre-specified. | 9  “*A priori* secondary outcomes including weight loss, GDM, gestational hypertension, pre-eclampsia, mode of delivery, length of stay in hospital, LGA, SGA, shoulder dystocia, and prematurity were extracted. When two or more studies compared the same cultural group for a given variable, they were analyzed and reported.”  13  “A subgroup analysis was conducted to compare differences between studies with large (N≥1000) or small (N<1000) cohorts” |
| **RESULTS** | | |  |
| Study selection | 17 | Give numbers of studies screened, assessed for eligibility, and included in the review, with reasons for exclusions at each stage, ideally with a flow diagram. | 9-10  “An outline of the study identification, inclusion and exclusion process is outlined in **Figure 1**. In total, 3,349 titles and abstracts were screened. Of these, 295 articles met the criteria for full text screening. Overall, 83 (78 unique samples) papers were identified as meeting the inclusion criteria and were included in the review.” |
| Study characteristics | 18 | For each study, present characteristics for which data were extracted (e.g., study size, PICOS, follow-up period) and provide the citations. | 10  “The majority of included articles (87%) were from studies conducted in North America (specifically the U.S.). Studies were also completed in Europe (9%), Asia (2%) and Africa (2%). Sample sizes ranged from 56(Shieh & Wu, 2014) to just over 600,000(Ki*m et a*l., 2014) women and included women ranging in age from under 20 to over 40 years, with most women being within the ages of 20-29 years. Articles that were quantitatively analyzed most frequently included the racial/ethnic groups of White/Non-Hispanic White (71%), Black/Non-Hispanic Black (65%), Hispanic (45%), and Asian (19%). Over half of the studies (64%) reported on nationality. Language (16%) and acculturation (16%) were the least reported indicators of culture. Our main outcome, GWG, was most often calculated with the use of self-reported pre-pregnancy BMI.” |
| Risk of bias within studies | 19 | Present data on risk of bias of each study and, if available, any outcome level assessment (see item 12). | 11  “A summary of the risk of bias assessments is presented in **Table S4.**  All studies had a low overall risk of bias; most bias originated from the use of convenience sampling (selection bias), or from self-reported pre-pregnancy weight (detection bias). Subsequently, selection bias and detection bias were examined by comparing studies with high vs. low bias, and changes greater than ±5% were noted (data not shown).” |
| Results of individual studies | 20 | For all outcomes considered (benefits or harms), present, for each study: (a) simple summary data for each intervention group (b) effect estimates and confidence intervals, ideally with a forest plot. | 11-12  “Overall, half of the women of White, Black, Hispanic and Asian racial/ethnic groups gained in excess of the current IOM guidelines (46%, 95% CI: 42%-55%, *I^2^*=0.0%; **Figure 2**). White women experienced excessive GWG most often (55%, 95% CI: 53%-57%, *I^2^*= 68.9%), and significantly more so than Asian (43%, 95% CI: 38%-47%, *I^2^*= 64.9%) and Hispanic women (46%, 95% CI: 45%-53%, *I^2^*= 68.9%); Black women had higher prevalence of excessive GWG (50%, 95% CI: 47%-53%, *I^2^* = 60.7%)” |
| Synthesis of results | 21 | Present results of each meta-analysis done, including confidence intervals and measures of consistency. | 12-17  “Overall, half of the women of White, Black, Hispanic and Asian racial/ethnic groups gained in excess of the current IOM guidelines (46%, 95% CI: 42%-55%, *I^2^*=0.0%; **Figure 2**). White women experienced excessive GWG most often (55%, 95% CI: 53%-57%, *I^2^*= 68.9%), and significantly more so than Asian (43%, 95% CI: 38%-47%, *I^2^*= 64.9%) and Hispanic women (46%, 95% CI: 45%-53%, *I^2^*= 68.9%); Black women had higher prevalence of excessive GWG (50%, 95% CI: 47%-53%, *I^2^* = 60.7%)” |
| Risk of bias across studies | 22 | Present results of any assessment of risk of bias across studies (see Item 15). | 11  “The Hispanic ethnic group was the only group of the four that experienced greater variability in this comparison, whereby high selection bias produced a high prevalence (38%) of excessive GWG than low bias (21%). Similarly, high detection bias reduced prevalence of excessive gain (20%) compared to low detection bias (47%). High selection bias or detection bias did not influence prevalence of inadequate GWG in either Black, White or Hispanic racial/ethnic groups.” |
| Additional analysis | 23 | Give results of additional analyses, if done (e.g., sensitivity or subgroup analyses, meta-regression [see Item 16]). | 11-17 |
| **DISCUSSION** | | |  |
| Summary of evidence | 24 | Summarize the main findings including the strength of evidence for each main outcome; consider their relevance to key groups (e.g., healthcare providers, users, and policy makers). | 17  “Regardless of which set of guidelines were used, a high proportion of studies (77%) reported some degree of influence of culture – whether that be race, ethnicity, language or immigration status – on achieving optimal GWG. Our findings show that White women were more likely to exceed the IOM guidelines than their Asian and Hispanic counterparts, but had a similar prevalence of exceeding guidelines to Black women.” |
| Limitations | 25 | Discuss limitations at study and outcome level (e.g., risk of bias), and at review-level (e.g., incomplete retrieval of identified research, reporting bias). | 20  “This study has several limitations. While we sought a globally representative sample, 87% of the articles meeting inclusion were conducted in North America (especially the U.S.), most of which compared a small number of racial/ethnic groups (Black, White, Hispanic and Asian). As such, this limits the generalizability of our results to other cultural subgroups and the strength of recommendations made by the IOM on a universal scale.” |
| Conclusions | 26 | Provide a general interpretation of the results in the context of other evidence, and implications for future research. | 21  “The large majority of women experienced discordant GWG, and this was consistently shown to be culturally dependent, wherein minority groups such as Black, Hispanic and Asian wom” |
| **FUNDING** | | |  |
| Funding | 27 | Describe sources of funding for the systematic review and other support (e.g., supply of data); role of funders for the systematic review. | On PeerJ Portal |

*From:*  Moher D, Liberati A, Tetzlaff J, Altman DG, The PRISMA Group (2009). Preferred Reporting Items for Systematic Reviews and Meta-Analyses: The PRISMA Statement. PLoS Med 6(6): e1000097. doi:10.1371/journal.pmed1000097

For more information, visit: **www.prisma-statement.org**.

Page 2 of 2
