# Supplementary material for: Addressing cultural, racial and ethnic discrepancies in guideline discordant gestational weight gain: a systematic review and meta-analysis
Source: PeerJ. 2018 Aug 27;6:e5407. doi: 10.7717/peerj.5407 (PMC6118200; doi:10.7717/peerj.5407)
Supplement: Supplemental Information 3 [file peerj-06-5407-s003.docx]

**Title:** Addressing cultural, racial and ethnic discrepancies in guideline discordant gestational weight gain: a systematic review and meta-analysis

**Authors:**

Kathryn M DENIZE, BSc^1^, Nina ACHARYA, Ms.^1^, Stephanie A PRINCE, PhD^2^, Danilo Fernandes DA SILVA, PhD^3^, Alysha LJ HARVEY, BSc^1^, Zachary M FERRARO, PhD^4^, Kristi B ADAMO, PhD*^1^

^1^ School of Human Kinetics, Faculty of Health Sciences, University of Ottawa, ON, Canada

^2^Division of Prevention and Rehabilitation, University of Ottawa Heart Institute, Ottawa, ON, Canada

^3^ Department of Physical Education, State University of Midwest/Parana (UNICENTRO), Guarapuava, PR 85040-080, Brazil.

^4^ Faculty of Medicine, University of Ottawa, Ottawa, ON, Canada

***Address of corresponding author:** Kristi B. Adamo, School of Human Kinetics, University of Ottawa, 200 Lees Avenue, Ottawa, ON, K1N 6N5, Canada. Email: [kadamo@uottawa.ca](mailto:kadamo@uottawa.ca). Phone: 613-562-5800 x1009.

**Keywords:** Culture, Ethnicity, Gestational Weight Gain, Meta-analysis, Pregnancy, Systematic Review.

**Acknowledgements:** The authors would like to thank the librarians Erica Wright and Mish Boutet, University of Ottawa, Ottawa, Canada, for their help with creating the search strategy. The authors would also like to recognize the students Mr Mitchell Crozier, Mr Jonathan Rankin, and Ms Ashley Weeks, University of Ottawa, Ottawa, Canada, for their help with data extraction. These students received stipends from their supervisor Kristi Adamo. Kathryn Denize holds the Canadian Graduate Scholarship – Masters Program, funded by Canadian Institute of Health Research. Kristi Adamo holds a Canadian Institute of Health Research New Investigator award. The findings reported were presented at the Canadian Obesity Summit, Banff, Alberta, Canada in Spring 2017.

**Conflicts of Interest:** The authors report no conflict of interest

**Table S1. Sample Ovid MEDLINE search strategy**

| 1 | Pregnancy/ |
| --- | --- |
| 2 | Pregnant women/ |
| 3 | Pregnancy outcomes/ or pregnancy complications/ |
| 4 | (Pregnan* or gestation*).ti,ab. |
| 5 | or/1-4 |
| 6 | Exp body weight changes |
| 7 | (Weight adj2 chang*).ti,ab. |
| 8 | (Weight adj2 (gain* or increase*)).ti,ab. |
| 9 | (Weight adj2 (loss or lose or losing or decrease*)).ti,ab. |
| 10 | ((BMI or body mass index) adj2 (increase* or decrease* or change* or loss*)).ti,ab. |
| 11 | or/6-10 |
| 12 | 5 and 11 |
| 13 | Culture/ |
| 14 | Acculturation/ |
| 15 | Cross-cultural comparison/ |
| 16 | Cultural characteristics/ |
| 17 | Cultural diversity/ |
| 18 | Cultural evolution/ |
| 19 | Exp ethnic groups/ |
| 20 | Exp continental population groups/ |
| 21 | (Acculturation* or sociocultural* or socio-culture* or cultural* or ethnic* or ancestry* or ethnoracial*).ti,ab. |
| 22 | or/13-23 |
| 23 | 12 and 22 |
| *different variations/forms of the term | |

**Table S2.** Study characteristics of studies that utilized alternative guidelines

| **First author, year** | **Country of study** | **N_analyzed_** | **Population description** | **Age (mean ± SD or range [%], years)** | **Racial/ethnic groups** |
| --- | --- | --- | --- | --- | --- |
| (Abrams, Carmichael, and Selvin 1995) | U.S. | 10,418 | General population | 28 (NR) | Asian, Hispanic, Black, White |
| (Ademowore, Courey, and Kime 1972) | U.S. | 345 | General population | Majority <21 years | White, Non-white |
| (Albers 1994) | U.S | 9,402 | General population | U.S.: <20 12.4%; 20-29 59.2%; >30 28.4% | Hispanic, Non-Hispanic |
|  |  |  |  | Southwest: <20 11.6%; 20-29 58.6%; >30 29.8% |  |
|  |  |  |  | New Mexico: <20 14.8%; 20-29 65.8%; >30 19.4% |  |
| (Allen et al. 1994) | Kenya | 3,011 | General population | Egyptian: 26.6 (NR) | Egyptian, Kenyan, Mexican |
|  |  |  |  | Kenyan: 30.6 (NR) |  |
|  |  |  |  | Mexican: 30.3 (NR) |  |
| (Bahadoer et al. 2015) | Netherlands | 6,444 | General population | Dutch: 31.2±4.5 | Dutch, Cape Verdeans, Dutch Antilleans, Moroccans, Surinamese-Creole, Surinamese-Hindustani, Turkish |
|  |  |  |  | Cape Verdean: 27.3±6.0 |  |
|  |  |  |  | Dutch Antillean: 26.3±5.5 |  |
|  |  |  |  | Moroccan: 28.1±5.3 |  |
|  |  |  |  | Surinamese-Creole: 27.9±6.5 |  |
|  |  |  |  | Surinamese-Hindustani: 27.6±4.9 |  |
|  |  |  |  | Turkish: 27.2±4.9 |  |
| (Bentley-Lewis et al. 2014) | U.S. | 1,043 | General population | No GDM: 30.3±6.1 | White, Black, Asian, Hispanic, Other |
|  |  |  |  | GDM: 32.2±5.4 |  |
| (L. Caulfield, Witter, and Stoltzfus 1996) | U.S. | 3,870 | Black or White women | Inadequate GWG: 24.1±6.2 | Black, White |
|  |  |  |  | Adequate GWG: 25.3±6.4 |  |
|  |  |  |  | Excessive GWG: 25.4±6.3 |  |
| (L. E. Caulfield, Stoltzfus, and Witter 1998) | U.S | 3,870 | Black or White women | Underweight: Black 21.7±4.8; White 27.1±6.6 | Hispanic, Black, White |
|  |  |  |  | Normal weight: Black 22.7±5.3; White 29.8±5.8 |  |
|  |  |  |  | Overweight: Black 24.9±6.0; White 28.2±5.5 |  |
| (Chasan-Taber et al. 2008) | U.S. | 770 | Self-identified Hispanic women | <20: 35.0% | Spanish, Non-Spanish |
|  |  |  |  | 20-24: 37.4% |  |
|  |  |  |  | 25-29: 17.9% |  |
|  |  |  |  | 20-29: 9.7% |  |
| (Ellerbe et al. 2013) | U.S. | 211,754 | General population | NWH: No DM 27.0±5.9; GDM 29.6±5.9 | NHW, NHB |
|  |  |  |  | NHB:  No DM 24.3±5.6;  GDM 27.5±6.2 |  |
| (Finch, Frank, and Hummer 2000) | U.S. | 13,247 | Oversampled Black infants and very low and low birth weight infants | 15-19: 12.8% | Black, White, Hispanic |
|  |  |  |  | 20-34: 79.4% |  |
|  |  |  |  | ≥35: 7.8% |  |
| (Frisbie, Forbes, and Hummer 1998) | U.S. | 9+ million | General population | <18: 3.04% | Five Hispanic groups (Mexican American, Puerto Ricans, Cubans, Central and South Americans, Other) |
|  |  |  |  | ≥18: 96.96% |  |
| (Romy Gaillard et al. 2013) | Netherlands | 6,959 | General population | Presented as Median (90% range) – 30.3 (20.4-37.9) | European vs. Non-European |
| (Hackley et al. 2010) | U.S. | 43 | Self-identified Hispanic women | Non-pregnant Nulliparous: 20.0±2.4 | Hispanic (compared English speaking vs. Spanish speaking) |
|  |  |  |  | Pregnant: 22.2±6.2 |  |
|  |  |  |  | Postpartum: 21.7±4.6 |  |
| (Hardy 1999) | U.S. | 213 | Women who were diagnosed with GDM | Age at GDM diagnosis: 28.51±5.57 | Black, White, Hispanic, Other (predominantly Asian) |
| (Heilemann et al. 2000) | U.S. | 773 | Self-identified Hispanic women | Mexico-oriented: 27±5.9 | Three Mexican groups (Born in Mexico/Spanish speaking, born in U.S./English speaking, born in Mexico/English speaking or born in U.S./Spanish speaking) |
|  |  |  |  | Intermediate: 2.5±6.0 |  |
|  |  |  |  | U.S.-oriented: 24.0±5.9 |  |
| (Hernandez-Rivas et al. 2013) | Spain | 456 | Women who were diagnosed with GDM | White: 33.37±5.21 | White, South Central Asians, Latin Americans, Moroccans, East Asians |
|  |  |  |  | Latin American: 31.38±5.35 |  |
|  |  |  |  | South-Central Asians: 31.72±6.03 |  |
|  |  |  |  | Moroccan: 33.92±4.85 |  |
|  |  |  |  | East Asian: 32.97±5.06 |  |
| (Hickey et al. 1990) | U.S. | 325 | General population | Black: 22.65±4.48 | Black, Hispanic |
|  |  |  |  | Hispanic: 23.18±4.78 |  |
| (Hickey et al. 1993)* | U.S. | 1,518 | High risk women | Black: 25.2±4.4 | Black, White |
|  |  |  |  | White: 24.8±4.4 |  |
| (Hickey et al. 1995a)* | U.S. |  | High risk women | Black: 24.8±4.3 | Black, White |
|  |  |  |  | White: 24.6±4.4 |  |
| (Hickey et al. 1995b)* | U.S. | 1,014 | High risk women | Black: 24.8±4.4 | Black, White |
|  |  |  |  | White: 24.4±4.4 |  |
| (Hickey et al. 1996)* | U.S. | 415 | High risk women of low & normal weight status | Black: 25.8±4.2 | Black, White |
|  |  |  |  | White: 25.2±4.3 |  |
| (Hickey et al. 1997a)* | U.S. | 806 | High risk women | Black: 24.8±4.3 | Black, White |
|  |  |  |  | White: 24.6±4.4 |  |
| (Hickey et al. 1997b) | U.S. | 5,918 | General population | Black: 23.7±5.0 | Black, White |
|  |  |  |  | White: 23.4±4.6 |  |
| (Hickey et al. 1999) | U.S. | 19,017 | General population | 18-20: 30.4% | Black, White |
|  |  |  |  | 21-25: 39.8% |  |
|  |  |  |  | 26-35: 26.8% |  |
|  |  |  |  | ≥36: 3.0% |  |
| (Huynh, Borrell, and Chambers 2014) | U.S. | 56, 911 | General population | 20-24: 38.6% | NHB, NHW, Hispanic |
|  |  |  |  | 25-29: 27.9% |  |
|  |  |  |  | 30-34: 22.2% |  |
|  |  |  |  | ≥35: 11.2% |  |
| (Koh et al. 2013) | Singapore | 1,166 | Chinese, Malay, or Indian women | ≤19: 2.3% | Chinese, Malay, Indian |
|  |  |  |  | 20-30: 51.0% |  |
|  |  |  |  | ≥31: 46.7% |  |
| (Margerison-Zilko et al. 2012) | U.S. | 3,070 | General population | 27.8±5.7 | Black, White, Other |
| (Misra, Hobel, and Sing 2010) | U.S. | 435 | General population | Black: <21 12.3%; 21-30 58.8%; >30 28.9% | Black, Non-Black |
|  |  |  |  | Non-Black: <21 9.3%; 21-30 51.2%; >30 39.5% |  |
| (Morling, Kitayama, and Miyamoto 2003) | Japan & U.S. | 158 | General population | U.S.: 29.4 (NR) | American, Japanese |
|  |  |  |  | Japan: 29.4 (NR) |  |
| (Neser 1963) | South Africa | 389 | General population | NR | Bantu, White (five groups: New York, Washington, Eugene, Ann Arbor, Jersey City, East Orange) |
| (Niswander et al. 1969) | U.S. | 15,204 | General population | NR | White, Black |
| (Ochsenbein-Kollble et al. 2007) | Switzerland | 4,034 | General population | White: 28.6±5.7 | White, Black, Asian |
|  |  |  |  | Asian: 29.3±5.0 |  |
|  |  |  |  | Black: 28.2±5.5 |  |
| (Petitti, Croughan-Minihane, and Hiatt 1991) | U.S. | 308 | General population | NR | White, Black |
| (Rosenberg et al. 2005) | U.S. | 373,325 | General population | NHB: 27.5 (NR) | NHB, NHW, NHA, Hispanic |
|  |  |  |  | NHW: 30.6 (NR) |  |
|  |  |  |  | NHA: 29.7 (NR) |  |
|  |  |  |  | Hispanic: 26.4 (NR) |  |
| (Sackoff and Yunzal-Butler 2014) | U.S. | 115,651 | General population | Black non-Hispanic: 24.3±5.2 | Black non-Hispanic, White non-Hispanic, Asian/Pacific Islander |
|  |  |  |  | Hispanic: 23.8±4.8 |  |
|  |  |  |  | White non-Hispanic: 28.1±5.4 |  |
|  |  |  |  | Asian/Pacific Islander: 27.3±4.5 |  |
| (Savitz et al. 2011) | U.S. | 3,872 | General population | NR | NHW, NHB, Hispanic, Asian |
| ( Schieve, Cogswell, and Scanlon 1998a) | U.S. | 126,605 | General population | Range 10-55; Majority 20-29 | NHB, NHW, Hispanic, Asian, Native American |
| (Schieve, Cogswell, and Scanlon 1998b) | U.S. | 173,066 | General population | NR | White, Black, Hispanic |
| (Sparks 2009) | U.S. | ~7,800 | General population | NHW: <20 5.17%; 20-34 74.84%; >34 19.99% | NHW, Mexico-born Mexican, U.S.-born Mexican, NHB, Native American, Asian, ‘Other’ Hispanics |
|  |  |  |  | Mexico-born Mexican: <20 7.83%; 20-34 74.84%; >34 13.43% |  |
|  |  |  |  | U.S.-born Mexican: <20 14.57%; 20-34 76.43%; >34 9.00% |  |
|  |  |  |  | NHB: <20 12.66%; 20-34 74.14%; >34 13.20% |  |
|  |  |  |  | Native American: <20 11.80%; 20-34 79.85%; >34 8.35% |  |
|  |  |  |  | Asian: <20 3.22%; 20-34 73.74%; >34 23.04% |  |
|  |  |  |  | ‘Other’ Hispanics: <20 8.99%; 20-34 77.39%; >34 13.62% |  |
| ( Stotland et al. 2005) | U.S. | 1,198 | General population | 18-23: 14.9% | Asian, Black, Latino, White |
|  |  |  |  | 24-29: 30.1% |  |
|  |  |  |  | 30-35: 37.6% |  |
|  |  |  |  | 36-47: 17.4% |  |
| ( Stotland et al. 2006) | U.S. | 15,101 | General population | White: 29.43 (NR) | White, Black, Latino, Asian |
|  |  |  |  | Black: 24.25 (NR) |  |
|  |  |  |  | Latina: 26.17 (NR) |  |
|  |  |  |  | Asian: 29.10 (NR) |  |
| (Taffel, Keppel, and Jones 1993) | U.S. | 9, 953 | General population | >15 | White, Blacks, |
| (Walker and Kim 2002) | U.S. | 305 | General population | White: 22.6±4.3 | White, Black, Hispanic |
|  |  |  |  | Black: 22.4±3.4 |  |
|  |  |  |  | Hispanic: 21.9±3.4 |  |
| (Wells et al. 2006) | U.S. | 4,944 | General population | 15-19: 11.2% | NHW, Hispanic, Black, Other |
|  |  |  |  | 20-24: 23.5% |  |
|  |  |  |  | 25-43: 51.1% |  |
|  |  |  |  | 35+: 14.2% |  |
| (Widen et al. 2015) | U.S | 302 | African American or Dominican women | Enrolled cohort with pregnancy weight-gain: 25.0±4.9 | Black, Dominican |
|  |  |  |  | Excluded because of loss to follow-up or missing covariate data: 25.6±5.9 |  |

NHB = non-Hispanic Black; NHW = non-Hispanic White; U.S. = United States; NR = not reported; DM = Diabetes; GDM = Gestational Diabetes Mellitus

**Table S3.** Pre-pregnancy BMI by racial/ethnic groups from articles using 2009 IOM guidelines, listed in alphabetical order by author

| **Author, year** | **Pre-pregnancy BMI, (kg/m^2^** **mean ± SD or classification [%])** | | | |
| --- | --- | --- | --- | --- |
|  | **NHW/White** | **NHB/Black** | **Hispanic** | **Asian** |
| (Badreldin et al. 2018) | Not reported | | | |
| (Berggren, Stuebe, and Boggess 2015) | Not reported | | | |
| (Bodnar et al. 2011) | Normal weight | Normal weight |  |  |
| (Bowers et al. 2013) | 24.6±5.7 | 27.4±7.3 | 25.5±5.6 |  |
| (Cavicchia et al. 2014) | UW: 5.1% | UW: 3.6% | UW: 3.5% |  |
|  | NW: 48.5% | NW: 33.7% | NW: 45.9% |  |
|  | OW: 24.0% | OW: 26.3% | OW: 31.3% |  |
|  | OB: 22.6% | OB: 36.5% | OB: 19.3% |  |
| (Chaffee et al. 2015) | 22.4±4.2 | 22.6±4.3 | 22.8±3.8 |  |
| (Chang et al. 2017) | Not reported | | | |
| (Cheng et al. 2015) | UW: 4.7% |  |  | UW: 10.4% |
|  | NW: 54.0% |  |  | NW: 69.6% |
|  | OW: 23.0% |  |  | OW: 15.5% |
|  | OB: 18.3% |  |  | OB: 4.4% |
| (Chihara et al. 2014) | Not reported | | | |
| (Cohen et al. 2016) | Not reported | | | |
| (Cox Bauer et al. 2016) | Not reported | | | |
| (Fontaine et al. 2012) | UW: 2% | UW: 3% |  |  |
|  | NW: 47% | NW: 33% |  |  |
|  | OW: 28% | OW: 30% |  |  |
|  | OB: 24% | OB: 34% |  |  |
| (Haile et al. 2017) | Not reported | | | |
| (Harris et al. 2015) | Not reported | | | |
| (I. Headen et al. 2015) | UW: 8.0% | UW: 7.0% | UW: 5.4% |  |
|  | NW: 68.0% | NW: 60.0% | NW: 63.9% |  |
|  | OW: 15.4% | OW: 20.0% | OW: 21.9% |  |
|  | OB: 8.6% | OB: 12.9% | OB: 6.8% |  |
| (Herring et al. 2008) | Not reported | | | |
| (Hunt et al. 2013) | UW: 0.0% | UW: 0.0% |  |  |
|  | NW: 34.3% | NW: 50.8% |  |  |
|  | OW: 27.1% | OW: 25.7% |  |  |
|  | OB: 38.6% | OB: 24.0% |  |  |
| (Kim et al. 2014) | UW: 5.3% | UW: 3.7% | UW: 3.6% | UW: 11.4% |
|  | NW: 54.7% | NW: 38.5% | NW: 51.4% % | NW: 67.4% |
|  | OW: 22.2% | OW: 27.6% | OW: 26.6% | OW: 16.1% |
|  | OB: 17.8% | OB: 30.2% | OB: 18.4% | OB: 5.2% |
| (Krukowski et al. 2013) | Not reported | | | |
| (Larouche et al. 2010) | Not reported | | | |
| (Leonard et al. 2017) | UW: 7.6% | UW: 6.6% | UW: 4.3% |  |
|  | NW: 66.8% | NW: 61.6% | NW: 64.8% |  |
|  | OW: 16.1% | OW: 19.9% | OW: 22.6% |  |
|  | OB: 9.5% | OB: 11.8% | OB: 8.3% |  |
| (Magriples et al. 2013) | UW: 6.1% | UW: 6.3% |  |  |
|  | NW: 43.4% | NW: 43.1% |  |  |
|  | OW: 20.9% | OW: 21.3% |  |  |
|  | OB: 25.0% | OB: 24.7% |  |  |
| (Mendez et al. 2014) | UW: 4.2% | UW: 3.6% |  |  |
|  | NW: 57.5% | NW: 43.5% |  |  |
|  | OW: 21.8% | OW: 26.8% |  |  |
|  | OB: 16.5% | OB: 26.0% |  |  |
| (Mendez et al. 2016) | UW: 4.1% | UW: 3.6% |  |  |
|  | NW: 57.1% | NW: 42.8% |  |  |
|  | OW: 21.9% | OW: 26.5% |  |  |
|  | OB: 16.9% | OB: 27.1% |  |  |
| (Pawlak et al. 2013) | UW: 4.3% | UW: 4.5% | UW: 3.9% |  |
|  | NW:53.4% | NW: 46.0% | NW: 45.5% |  |
|  | OW: 23.1% | OW: 27.1% | OW: 28.1% |  |
|  | OB:17.7% | OB: 22.7% | OB: 22.5% |  |
| (Shieh and Wu 2014) |  | 28±6.9 | 26.2±4.8 |  |
| (Sridhar et al. 2014) | Not reported | | | |
| (Torloni et al. 2012) | UW: 4.7% | UW: 2.4% |  |  |
|  | NW: 46.8% | NW: 36.6% |  |  |
|  | OW: 25.1% | OW: 25.8% |  |  |
|  | OB: 23.4% | OB: 35.2% |  |  |

UW = underweight, NW = normal weight, OW = overweight, OB = obese

**Table S4.** Summary of how GWG was calculated in studies that utilized the 2009 IOM guidelines

| **First author, year** | **Gestational Weight Gain** |
| --- | --- |
| (Badreldin et al. 2018) | Weight at delivery minus pre-pregnancy weight |
| (Berggren, Stuebe, and Boggess 2015) | Weight at last prenatal visit (≥ 35 weeks gestation and within 2 weeks of delivery) minus pre-pregnancy weight or first prenatal visit weight (<20 weeks gestation) |
| (Bodnar et al. 2011) | Weight at last prenatal visit minus pre-pregnancy weight |
| (Bogaerts et al. 2012) | Weight at delivery minus pre-pregnancy weight |
| (Bowers et al. 2013) | Obtained from patient electronic medical records |
| (Cavicchia et al. 2014) | Obtained from birth certificates |
| (Chaffee et al. 2015) | Weight at delivery minus pre-pregnancy weight |
| (Chang et al. 2017) | Obtained from medical rcords |
| (Chasan-Taber et al. 2016a) | Last weight prior to delivery minus pre-pregnancy weight |
| (Cheng et al. 2015) | Body weight at birth minus pre-pregnancy weight |
| (Chihara et al. 2014) | Weight at delivery minus pre-pregnancy weight |
| (Cohen et al. 2016) | Weight at delivery minus pre-pregnancy weight |
| (Cox Bauer et al. 2016) | Weight at admission prior to delivery minus pre-pregnancy weight |
| (Deputy et al. 2015) | Obtained from birth certificate |
| (Fontaine et al. 2012) | Last measured prenatal weight (did not define time point) minus baseline weight (did not define when ‘baseline’ was) |
| (Rothberg et al. 2011) | Weight at last prenatal care visit minus pre-pregnancy weight |
| (Haile et al. 2017) | Weight at delivery minus pre-pregnancy weight |
| (Harris et al. 2015) | Weight at delivery minus pre-pregnancy weight |
| (I. Headen et al. 2015) | Weight at delivery minus pre-pregnancy weight. When a woman delivered prior to term, GWG adequacy was calculated (an estimated ratio of a woman's expected and observed amounts of weight gain at each week of gestation |
| (Hedderson and Ferrara 2010) | Weight at glucose screening test minus pre-pregnancy weight, divided by the weeks of gestation at the time of the screening test (did not measure total GWG) |
| (Herring et al. 2008) | Last measured weight prior to delivery (mean 1.2 weeks before gestation) minus first measured weight during early pregnancy (<14 weeks gestation) |
| (Hunt et al. 2013) | Weight at delivery minus pre-pregnancy weight |
| (Kim et al. 2014) | Weight at delivery minus pre-pregnancy weight |
| (Kinnunen et al. 2016) | Information on total GWG was self-reported at a visit at 14 weeks postpartum (did not explain calculation) |
| (Koleilat and Whaley 2013) | Weight at last clinic visit (1 month or less of delivery date) minus weight at first trimester (did not define time point) |
| (Kowal, Kuk, and Tamim 2012) | Obtained from Canadian Maternity Experience Survey |
| (Krukowski et al. 2013) | Derived from a data set (did not explain calculation) |
| (Larouche et al. 2010) | Extrapolated total GWG from weight gain documented between the first and last prenatal visits (did not define time point) |
| (Leonard et al. 2017) | Weight at delivery minus pre-pregnancy weight |
| (Lindberg et al. 2016) | Last weight prior to delivery minus pre-pregnancy weight |
| (Magriples et al. 2013) | Weight recorded during third trimester of pregnancy (mean = 34.5 weeks’ gestation) minus pre-pregnancy weight |
| (Mendez et al. 2014) | Weight at delivery minus pre-pregnancy weight |
| (Mendez et al. 2016) | Weight at delivery minus pre-pregnancy weight |
| (Pawlak et al. 2013) | Weight at delivery minus pre-pregnancy weight |
| (Shieh and Wu 2014) | Weight at time of data collection (did not define time point; cross-sectional data collection) minus pre-pregnancy weight |
| (Sommer et al. 2014) | Weight at second visit during gestation (28 ± 2 weeks) minus weight at first visit during gestation (<20 weeks gestation, mean =12 (3)) |
| (Sridhar et al. 2014) | Last measured pregnancy weight (did not define time point) minus pre-pregnancy weight (recorded 12 months before pregnancy) |
| (Torloni et al. 2012) | Weight at delivery minus pre-pregnancy weight |
| (Tovar et al. 2012) | Weight at delivery minus pre-pregnancy weight |
| (Walker, Cheng, and Brown 2014) | Weight at the end of pregnancy (did not define time point) minus pre-pregnancy weight |

**Table S5.** Risk of Bias assessment, using the Modified Cochrane Tool, of studies that used the 2009 IOM guidelines

| **First author, year** | **Selection Bias** | **Performance Bias** | **Detection Bias** | **Selective Reporting Bias** | **Attrition Bias** | **Other Bias** |
| --- | --- | --- | --- | --- | --- | --- |
| (Badreldin et al. 2018) | High | Low | Unclear | Unclear | Unclear | Unclear |
| (Berggren, Stuebe, and Boggess 2015) | High | Low | Unclear | Unclear | Unclear | Low |
| (Bodnar et al. 2011) | High | Low | Low | Low | Low | Low |
| (Bogaerts et al. 2012) | High | Low | High | Low | Unclear | High |
| (Bowers et al. 2013) | High | Low | Low | Low | Unclear | Low |
| (Cavicchia et al. 2014) | High | Low | Low | Low | Low | Low |
| (Chaffee et al. 2015) | Low | Unclear | Low | Low | Low | Low |
| (Chang et al. 2017) | Low | Unclear | High | Unclear | Unclear | High |
| (Chasan-Taber et al. 2016a) | High | Low | High | High | Low | Low |
| (Cheng et al. 2015) | Low | Low | High | Unclear | Low | Low |
| (Chihara et al. 2014) | High | Low | Low | Unclear | Unclear | Low |
| (Cohen et al. 2016) | Low | Low | High | Unclear | Unclear | Low |
| (Cox Bauer et al. 2016) | Low | Low | Unclear | Low | Low | Low |
| (Deputy et al. 2015) | Low | Low | High | Low | High | Low |
| (Fontaine et al. 2012) | Low | Low | Low | High | Low | Low |
| (Rothberg et al. 2011) | Low | Low | High | Unclear | Low | Low |
| (Haile et al. 2017) | High | Unclear | Low | Unclear | Unclear | Low |
| (Harris et al. 2015) | Low | Low | High | Low | Low | Low |
| (I. Headen et al. 2015) | Low | Low | High | Unclear | High | High |
| (Hedderson, EP, and Ferrara 2010) | Low | Low | Low | Unclear | Low | High |
| (Herring et al. 2008) | High | Low | High | Low | Low | Low |
| (Hunt et al. 2013) | Low | Low | Low | Unclear | High | Low |
| (Kim et al. 2014) | Low | Low | High | Unclear | Unclear | Low |
| (Kinnunen et al. 2016) | Low | Low | Low | Low | Low | Low |
| (Koleilat and Whaley 2013) | Low | Low | Low | Unclear | Unclear | High |
| (Kowal, Kuk, and Tamim 2012) | Low | Low | High | Low | High | Low |
| (Krukowski et al. 2013) | Low | Low | High | Low | High | Low |
| (Larouche et al. 2010) | Low | Low | High | Unclear | Unclear | High |
| (Leonard et al. 2017) | Low | Unclear | High | Unclear | Low | High |
| (Lindberg et al. 2016) | Low | Low | High | Unclear | Unclear | High |
| (Magriples et al. 2013) | High | Low | Low | Low | Low | High |
| (Mendez et al. 2014) | Low | High | High | Unclear | Low | Low |
| (Mendez et al. 2016) | Low | Low | High | Low | Low | Low |
| (Pawlak et al. 2013) | Low | Low | High | Unclear | Unclear | Low |
| (Shieh and Wu 2014) | High | Low | Low | Low | High | High |
| (Sommer et al. 2014) | Low | Low | High | Low | Low | Low |
| (Sridhar et al. 2014) | Low | Low | High | Low | Unclear | Low |
| (Torloni et al. 2012) | Low | Low | High | Low | Unclear | Low |
| (Tovar et al. 2012) | High | Low | High | Low | High | High |
| (Walker, Cheng, and Brown 2014) | Low | Low | Low | Low | Low | Low |

**Table S6.** Main findings from articles using alternative guidelines, listed in alphabetical order by author

| **First author, year** | **Main Outcome** | **Summary of GWG Results** |
| --- | --- | --- |
| (Abrams, Carmichael, and Selvin 1995) | Rate of GWG per trimester | - Compared to NHW women, NHB women gained significantly faster during first trimester and slower during last two trimesters - Hispanic women gained more slowly than NHW women during the first two trimesters and significantly faster during the third |
| (Ademowore, Courey, and Kime 1972) | Mean maternal weight gain | - Non-white women had lower GWG than white counterparts |
| (Albers 1994) | Low birth weight infants | - New Mexico women had a 10% higher mean GWG than other American women - In the whole USA, no significant difference in GWG between Hispanic and non-Hispanic women - In New Mexico, Hispanic women had an 88.2% chance of gaining over 25lb compared to 69.0% for non-Hispanics |
| (Allen et al. 1994) | BMI | - GWG did not differ by country/nationality (Mexican, Kenyan, Egyptian) |
| (Bahadoer et al. 2015) | Pre-pregnancy obesity and GWG | - Compared to Dutch-origin women, Surinamese-Hindustani-origin women (OR:0.40) and Moroccan-origin women (OR: 0.48) had lower risks of EGWG - Compared to Dutch-origin women, total GWG was lower in all ethnic minority groups, except for Cape Verdean-origin and Surinamese-Creole-origin women (*p*-values <0.05). |
| (Bentley-Lewis et al. 2014) | Hypertension | - A relationship between GWG and ethnicity existed across entire study population |
| (Caulfield, Witter, and Stoltzfus 1996) | GWG | - Black women were at increased risk of under-gain compared with white women - Black women were as likely as white women to have adequate GWG |
| (Caulfield, Stoltzfus, and Witter 1998) | Risk of delivering LGA or SGA infant | - Within each BMI stratum, black women were more likely to gain less total weight than white women |
| (Chasan-Taber et al. 2008) | GWG | - Women with < 10 yrs of residence in the United States were 50% less likely to gain above the IOM range as compared to third-generation women (95% CI o.3, 0.9). - Among Hispanic women, the prevalence of excessive GWG was higher in the English-only speakers compared to the English and Spanish speakers and Spanish-only speakers. |
| (Ellerbe et al. 2013) | Upper quartiles of birth weight | - NHW women without GDM gained slightly more weight on average than NHB with and without GDM and NHW women with GDM. |
| (Finch, Frank, and Hummer 2000) | Adverse birth outcomes (infant mortality) | - NHB women were more likely than NHW women to gain <15 pounds - NHB women had the highest mean GWG |
| (Frisbie, Forbes, and Hummer 1998) | Adverse birth outcomes (infant mortality, low birth weight, and prematurity) | - Among American Hispanics, Mexican Americans and Puerto Ricans have the lowest GWG, Anglo and Cuban Hispanics have the highest GWG |
| (Romy Gaillard et al. 2013) | EGWG | - European ethnicity had higher risk of EGWG than non-European women |
| (Hackley et al. 2010) | GWG and post-partum weight loss | - Spanish-speaking women had better adherence to GWG guidelines, however, language was not statistically associated with achieving GWG guidelines |
| (Hardy 1999) | Macrosomia | - Black women had higher mean GWG than White and Hispanic counterparts - White women had higher mean GWG than Hispanic counterparts |
| (Heilemann et al. 2000) | Prenatal and labour outcomes | - U.S born women of Mexican descent were more likely to have EGWG than Mexican-born women - Mexican-born women were more likely to have IGWG |
| (Hernandez-Rivas et al. 2013) | GDM | - GWG did not differ across cultures |
| Hickey, 1990(C A Hickey et al. 1990) | Weight for Height Z scores | - GWG did not differ by ethnicity |
| Hickey, 1993*(C. Hickey et al. 1993) | Birth weight | - Similar proportions of black and white women had IGWG |
| Hickey et al. 1995a*(C A Hickey et al. 1995) | Low prenatal weight gain | - 33-40% of black and white women had IGWG |
| Hickey, 1995b*(C. Hickey et al. 1995) | Spontaneous preterm delivery | - A wide range in total GWG was observed among women in both ethnic groups |
| Hickey, 1996*(Carol A. Hickey et al. 1996) | Birth weight | - GWG did not differ by race-ethnicity |
| Hickey, 1997a*(C A Hickey et al. 1997) | Low GWG | - 27.0% of low-income black women gained less than or equal to 10kg vs. 22.2% for low income white women - Low income black women gained less than low income white women |
| Hickey, 1997b(Carol A. Hickey et al. 1997) | Prenatal weight gain in the upper and lower halves of the recommended range of gain | - Approximately half of the women whose weight gain was within the recommended ranges had gains in the lower portion of the recommended range, regardless of ethnicity - The incidence of low prenatal weight gain was highest among black women and among women with low pregravid BMI in both ethnic groups - The incidence of high prenatal weight gain was highest among white women and among women with high pregravid BMI in both ethnic groups.\ |
| Hickey, 1999(C A Hickey et al. 1999) | Low GWG | - The incidence of low weight gain was increased among Black women who had < 12 yrs of education, were single, anemic, had low or normal pre-pregnancy body mass index, increased parity, interpregnancy intervals < 24 months, used tobacco or alcohol or entered prenatal care or WIC programs after the first trimester. |
| Huynh, 2013(Huynh, Borrell, and Chambers 2014) | EGWG | - NHB and Hispanic women were more likely to have EGWG than their NHW counterparts |
| Koh, 2013(Koh et al. 2013) | Discordant GWG | - Malay ethnicity was associated with higher EGWG and inadequate GWG in comparison to Chinese women |
| Margerison, 2012(Margerison-Zilko et al. 2012) | Birth weight for gestational age and BMI at age 5 | - Black women had higher GWG throughout pregnancy |
| Misra, 2010(Misra, Hobel, and Sing 2010) | Variation and rate of weight gain | - The rate of weight gain in the first half of pregnancy for African-American women was an average of 0.30 kg/week compared to 0.24 kg/week for non-African-American women |
| Morling, 2003(Morling, Kitayama, and Miyamoto 2003) | Coping strategies | - American women gained significantly more (mean 16.2 kg) than Japanese women (mean 9.6 kg) p < 0.0001 |
| Neser, 1963(Neser 1963) | GWG | - The average GWG of the Bantu women did not differ significantly from the literature values for white women on a rigidly restricted diet |
| Niswander, 1969(Niswander et al. 1969) | Birth weight | - The average GWG for black and white women did not differ (22.8 lb vs. 22.5 lb) - Black women had more inadequate and excessive GWG than white women |
| Ochsenbein-Kolbe, 2007(Ochsenbein-Kollble et al. 2007) | GWG | - Asian and Black women had consistent and significantly lower GWG compared to White women |
| Petitti, 1991(Petitti, Croughan-Minihane, and Hiatt 1991) | GWG | - GWG did not differ by race |
| Rosenberg, 2005(Rosenberg et al. 2005) | Adverse birth outcomes (caesarean section, preterm birth, LBW) | - NHB (20.3%) and Hispanic (20.9%) women were more likely to exhibit EGWG than NHW (16.8%) and Asian women (10.8%) |
| Sackoff, 2015(Sackoff and Yunzal-Butler 2014) | Interconception weight change | - NHW, NHB and Hispanic women had similar mean GWG (32.1 lb – 32.9 lb) and rates of EGWG (27-29%) - Asian/Pacific Islander has significantly lower mean GWG (30.2 lb) and rate of EGWG (18.0%) |
| Savitz, 2011(Savitz et al. 2011) | Association between GWG, preterm birth, SGA, LGA and birth method | - GWG did not differ by race |
| Schieve, 1998a(L. Schieve, Cogswell, and Scanlon 1998) | GWG | - Discordant GWG by culture changed between 1990-1996 - White women had the highest amount of GWG from 1990-1994, however, Native American women had the highest from 1995-1996. |
| Schieve, 1998b(L. A. Schieve, Cogswell, and Scanlon 1998) | Discordant GWG | - White women were less likely to report IGWG and more likely to report EGWG - White women averaged higher GWG than their black and Hispanic counterparts - GWG among obese women was similar across race-ethnicity groups |
| Sparks, 2009(Sparks 2009) | LBW | - U.S. born, Hispanic women are more at risk for EGWG than foreign born, Hispanic women. - NHW women had the highest odds of EGWG |
| Stotland, 2005(N. E. Stotland et al. 2005) | GWG by pre-pregnancy BMI | - Black and Latina women were more likely than white women to report target weight gain below the IOM guidelines, even when controlling for education status |
| Stotland, 2006(N. Stotland et al. 2006) | Spontaneous preterm birth | - Black women were most likely to gain less than 0.27kg/wk - % of women gaining above 0.52kg/wk lowest for Asians (13.8%), higher for white (24.6%) and African American (23.0%) |
| Taffel, 1993(Taffel, Keppel, and Jones 1993) | Prenatal care provider advice on GWG | - African American women were more likely to have inadequate GWG than White women |
| Walker, 2002(Walker and Kim 2002) | Psychosocial thriving | - GWG did not significantly differ by race |
| Wells, 2006(Wells et al. 2006) | Discordant GWG | - Hispanic women were more likely to have IGWG - NHW and NHB women were positively associated with EGWG |
| Widen, 2015(Widen et al. 2015) | Long term fat and weight retention | - EGWG did not differ by race-ethnicity |

*=used same data set. Abbreviations: BMI = body mass index, EGWG = excessive gestational weight gain; GWG = gestational weight gain, IGWG = inadequate gestational weight gain; IOM = Institute of Medicine; NHB = Non-Hispanic Black, NHW = non-Hispanic White, OB = obese, OW = overweight; OR = Odds ratio; WIC = Special Supplemental Nutrition Program for Women, Infants and Children; U.S = United States
